# Pulmonary Consequences of Prenatal Inflammatory Exposures: Clinical Perspective and Review of Basic Immunological Mechanisms

**DOI:** 10.3389/fimmu.2020.01285

**Published:** 2020-06-19

**Authors:** Courtney M. Jackson, Shibabrata Mukherjee, Adrienne N. Wilburn, Chris Cates, Ian P. Lewkowich, Hitesh Deshmukh, William J. Zacharias, Claire A. Chougnet

**Affiliations:** ^1^Division of Immunobiology, Cincinnati Children's Hospital Research Foundation, Cincinnati, OH, United States; ^2^Immunology Graduate Program, University of Cincinnati College of Medicine, Cincinnati, OH, United States; ^3^Division of Pulmonary and Critical Care Medicine, Department of Internal Medicine, University of Cincinnati, Cincinnati, OH, United States; ^4^Department of Pediatrics, University of Cincinnati College of Medicine, Cincinnati, OH, United States; ^5^Division of Neonatology/Pulmonary Biology, The Perinatal Institute, Cincinnati Children's Hospital Medical Center, University of Cincinnati, Cincinnati, OH, United States

**Keywords:** chorioamnionitis, fetal lung, fetal inflammatory response, fetal immunity, immune ontogeny

## Abstract

Chorioamnionitis, a potentially serious inflammatory complication of pregnancy, is associated with the development of an inflammatory milieu within the amniotic fluid surrounding the developing fetus. When chorioamnionitis occurs, the fetal lung finds itself in the unique position of being constantly exposed to the consequent inflammatory meditators and/or microbial products found in the amniotic fluid. This exposure results in significant changes to the fetal lung, such as increased leukocyte infiltration, altered cytokine, and surfactant production, and diminished alveolarization. These alterations can have potentially lasting impacts on lung development and function. However, studies to date have only begun to elucidate the association between such inflammatory exposures and lifelong consequences such as lung dysfunction. In this review, we discuss the pathogenesis of and fetal immune response to chorioamnionitis, detail the consequences of chorioamnionitis exposure on the developing fetal lung, highlighting the various animal models that have contributed to our current understanding and discuss the importance of fetal exposures in regard to the development of chronic respiratory disease. Finally, we focus on the clinical, basic, and therapeutic challenges in fetal inflammatory injury to the lung, and propose next steps and future directions to improve our therapeutic understanding of this important perinatal stress.

## Introduction

The epithelial surface of the lung is constantly exposed to external noxious stimuli, including pathogens, particulates, and toxins. To maintain function, the lung must be able to protect itself against or adequately respond to such insults. Failure of these defense mechanisms contributes to the development of disease, with potentially significant morbidity and/or mortality; in fact, respiratory diseases account for a substantial portion of the health burden faced by the global community (1–3). Though the incidence and prevalence of various diseases affecting the lung change with age, lung illnesses occur in every stage of life. Exposure of the lung to infectious and inflammatory insults during both childhood and adulthood is well-known to cause chronic lung disease, but less well-recognized is the significant impact of exposure during the prenatal period *in utero*, when critical lung development processes are ongoing (4). Indeed, the developing mammalian lung is constantly exposed to amniotic fluid, and modifications in the amount or character of this fluid can lead to severe abnormalities in lung development (5, 6). The lung epithelium is so intimately exposed to amniotic fluid that early experiments have been successful in using intra-amniotic (IA) delivery to perform epithelial genome editing (7). A growing body of evidence suggests that many fetal exposures influence lung development, and that these exposures impact the trajectory of lung function changes in adolescence and adulthood, the risk of future respiratory disease, and how the lung responds to future injury.

This review seeks to summarize the evidence supporting the role of fetal lung inflammation on lifelong respiratory dysfunction through the prism of chorioamnionitis, a highly prevalent and extensively studied fetal injury. Herein we will review the clinical characteristics, mechanistic data, and experimental models underlying our current understanding of chorioamnionitis. We provide in depth review of the immunological consequences and discuss strengths and weaknesses of available animal models. Finally, we identify key future directions with the goal of furthering our understanding of fetal inflammation and *in utero* injuries to the lung. Our hope is that defining the key pathways and open questions will help optimize future translational science to provide better understanding of chorioamnionitis and other fetal inflammatory stressors, ultimately leading to improved therapies for affected patients.

## Fetal Origins of Lung Disease

It has been classically taught that lung function gradually declines throughout life for all individuals (8). However, this analysis assumed a common starting point for all adults and ignored exposures prior to the age of 25. In particular, as the perinatal period represents a still developing system undergoing proscribed, sequential changes leading to complete maturation, exposures occurring during this period afford more opportunity for injury-induced alterations than exposures that occur in the more static, homeostatic systems found in most organs later in life. This idea, termed the “fetal origins of disease” hypothesis, is supported by studies demonstrating that experiences early in life, including the antenatal period, impact a variety of adult health metrics (9–16) including lung function (17–21). Furthermore, the same injury occurring in this early life period may have a more dramatic impact than the same exposure occurring later in life, simply because they occur during an early developmental time point. This concept is supported broadly by the literature of developmental knockouts in mice, where germline or early knockouts of many genes leads to global, and often severe, phenotypes in affected animals. In humans, the fetal origins hypothesis may also explain the observation that individuals with similar genotypes can have widely variable manifestations of disease secondary to timing of injuries or environmental events. Such differential presentations may subsequently result in the clinical impression of sporadic disease, complex risk profiles for disease progression, or incomplete penetrance of clinical phenomena. One clear example of such time-related phenotypes has been described for the metabolic disease phenylketonuria (PKU). It is known that infants exposed to elevated phenylalanine levels *in utero* due to maternal PKU are at high risk for injury, especially severe neurologic impairment, regardless of their genetic profile (22–25). Similarly, those infants who are born with PKU have poor neurologic outcomes if their disease is not recognized and treated early (26). However, in those infants with PKU who are treated with dietary modification early in life, the consequences of non-adherence to therapy later in life present only with subtle cognitive impairments (27, 28). Thus, it appears the timing of exposure to high phenylalanine levels, rather than the presence of exposure, is of key importance in determining phenotype. It is likely that many diseases with presentation in the perinatal period are impacted by timing and dose effects in a similar manner.

In accordance with these data, and as predicted by the fetal origins of disease hypothesis, a number of retrospective cohort studies demonstrate lifelong risk for the development of cardiovascular, metabolic, respiratory, and other disease (29–36) following significant early life stressors, namely famine. Subsequent cohort studies focused specifically on lung disease suggest substantial connections between early life exposures and the development of adult lung illnesses, particularly chronic obstructive pulmonary disease and asthma (18, 37–40). Prenatal, perinatal, and childhood factors that are associated with worse respiratory outcomes during childhood include biomass fuel exposure (41, 42), tobacco exposure (42–45), air pollution (42, 46), preterm birth (46–50), and respiratory tract infections (46, 51), among others. Many of these same factors are similarly associated with adult lung function (52–54), suggesting that antenatal and perinatal factors during lung development can have lifelong impact. These exposures are thought to impact respiratory outcomes through direct effects as well as, and potentially more significantly, the provocation of an inflammatory response within the lung. We now turn to chorioamnionitis, a well-studied model of fetal inflammatory stress, to examine how *in utero* exposure to inflammation impacts the developing lung.

## Pathological Definition of Chorioamnionitis

Chorioamnionitis is a technical, histopathologic term used to indicate inflammation of the placenta, specifically the chorion, amnion, or both (55). Up to 25–40% of preterm births are associated with chorioamnionitis (56, 57), and in very preterm infants (~24 weeks gestation), the incidence of chorioamnionitis can reach over 90% (58, 59). In severe cases, the inflammation can include additional structures, namely the umbilical cord. In the event of umbilical cord involvement, this inflammation is alternatively referred to as funisitis (59–61). Chorioamnionitis is classified according to both the qualitative degree of neutrophil infiltration within the placental membranes (grade 1–3) as well as the progression of neutrophil infiltration through the placental membranes (stage 1–3) utilizing the Redline criteria (61). Stage 1 and grade 1 chorioamnionitis are considered mild, whereas severe chorioamnionitis is defined by grade or stage >1 or the combination of chorioamnionitis and funisitis (61).

Importantly, histological chorioamnionitis is frequently clinically silent, with minimal maternal inflammatory response (62, 63). This is distinct from microbial invasion of the amniotic cavity, when culturable microorganisms are identified from amniotic fluid samples, and the placental and amniotic fluid inflammation is generally more severe (64). This is also different from the clinical diagnosis of chorioamnionitis, which is defined by manifestations such as intrapartum fever, maternal or fetal tachycardia, purulent or foul-smelling amniotic fluid or vaginal discharge, uterine tenderness, and maternal leukocytosis (65–68). In general, this review considers the literature with respect to the histological diagnosis, which is present in the majority of cases of *clinical* chorioamnionitis.

## Pathogenesis of Chorioamnionitis

The pathogenesis of chorioamnionitis has been a subject of investigation for decades. Initial hypotheses suggested microbial invasion of the amniotic cavity as the primary etiology (69). However, multiple subsequent studies have revealed that histological chorioamnionitis is often found in the absence of demonstrable infection (70, 71). Framed as “sterile intra-amniotic inflammation,” chorioamnionitis is induced by some yet to be determined danger signal (72–74). Notably, bacterial colonization of the amniotic fluid without significant resulting inflammation has not been associated with negative effects (75). Conversely, inflammation without detection of bacteria has been associated with adverse clinical outcomes similar to those seen with the combination of inflammation and bacteria (75). These results strongly imply that chorioamnionitis is best understood as a severe inflammatory response in the amniotic space, rather than the reaction to a specific infectious agent, and that this inflammation, regardless of etiology, is the proximate cause of much of the morbidity and mortality seen in clinical chorioamnionitis.

## Microbiology of Chorioamnionitis

Initial studies of the placental microbiome in subjects with severe chorioamnionitis showed particularly high abundance of urogenital and oral bacteria (notably *Ureaplasma parvum, Fusobacterium nucleatum* and *Streptococcus agalactiae)* and low levels of Lactobacilli (76). Further studies confirmed presence of urogenital and oral species, demonstrating strong correlation between severe chorioamnionitis and the presence of bacterial species, though specific species differ between studies (75, 77, 78). Using new techniques to study the microbiome, recent reports suggest microbial species diversity may be relevant, with diminished diversity in the placental membranes in severe chorioamnionitis compared to either mild chorioamnionitis or controls (76, 79). Severe chorioamnionitis was also associated with a significantly increased 16S rDNA copy number (79), suggesting a more robust infiltration of bacterial species overall. Nevertheless, these observations have been challenged by recent studies which found no distinction between negative background controls and placenta samples, even those from preterm births (80). A more recent study failed to detect any distinctive bacterial signature in placentas from cases of chorioamnionitis (81). While, *Ureaplasma* and *Mycoplasma* could be detected in the 16S rRNA gene sequence data from a small minority of preterm samples, these organisms were also usually detectable in vaginal swab samples from the same women, leaving it unclear whether these sequences originated from the placenta specimen or vaginal contamination during delivery.

## Chorioamnionitis and Postnatal Human Lung Function

An extensive literature documents the relationship of chorioamnionitis and lung function in the post-natal period. Initial studies identified a reduced risk of respiratory distress syndrome (RDS) but an increased risk of bronchopulmonary dysplasia (BPD) in preterm infants with chorioamnionitis (82). Tracheal lavage showed increases in inflammatory mediators including IL-1 in patients that developed BPD (83). Therefore, it was hypothesized that inflammation resulted in accelerated lung maturation, which explained decreased RDS, but had more long-term deleterious consequences on lung development, leading to increased risk of BPD. Since that time, multiple studies have tried to better delineate the relationship between chorioamnionitis, RDS, and BPD with mixed results. Multiple studies have confirmed the initial reports (68, 84–89), while recent meta-analyses have questioned the linearity of these relationships (90). A challenge in truly identifying the relationship between these pathologies is the lack of clarity in the ontogeny, diagnosis, classification, and treatment for each these disorders (91). Other confounding factors including gestational age and co-morbidities in the preterm population can also make these relationships difficult to quantify (91).

Supporting the impact of fetal inflammation on lung development, however, is the observation that exposure to the inflammatory state of severe chorioamnionitis is associated with adverse pulmonary outcomes in early childhood. In particular, a birth cohort followed through 2 years of age identified a strong joint effect of prematurity and chorioamnionitis on the risk of wheezing and asthma (92). This association may also partially drive the observed correlation between prematurity and wheezing and early life asthma seen in a subsequent meta-analysis of 31 different birth cohorts, though chorioamnionitis prevalence was not specifically reported (93). Additionally, a separate cohort demonstrates that exposure to severe chorioamnionitis, but not mild chorioamnionitis, is independently associated with wheeze and respiratory-related physician visits in the first year of life (94), suggesting that the degree of inflammatory injury may be directed related to outcomes.

While there are no currently available studies that explicitly describe a connection between chorioamnionitis and late childhood, adolescent, or adult lung function, there are studies that demonstrate an association between coincident factors, namely BPD and prematurity, and later lung function. Prematurity has been variably associated with increased respiratory symptoms, airflow obstruction, and airway hyperresponsiveness into early adulthood (95–98). Similarly, BPD has been associated with airflow obstruction, increased medication use, and respiratory symptoms in childhood, adolescence, and early adults (99–103). Unfortunately, studies have not determined whether either of these two conditions contribute to accelerated lung function decline or more severe, deleterious responses to future noxious stimuli. However, it is likely that the observed reduction in peak lung function contributes to the emergence of chronic or classical adult symptoms earlier in life.

## Lung Immune Milieu in Human Chorioamnionitis

Despite the challenges in precisely correlating chorioamnionitis to specific lung diseases, it is clear that an *in utero* inflammatory environment impacts the development of the lung and predisposes later lung dysfunction. Our understanding of the mechanisms driving these epidemiological associations is limited, though several molecules have been implicated in lung injury in patients. Severe granulo-histiocytic infiltration (104, 105) and an increase in apoptotic cells (106) are found in chorioamnionitis- exposed infant's lungs at autopsy. During chorioamnionitis, the concentration of inflammatory mediators including IL-1β, IL-6, IL-8, MMP9 and TNFα in the amniotic fluid increases dramatically (82, 107–111). Immune cell numbers, especially neutrophils, are also more abundant in amniotic fluid (112–114). It is suggested that these amniotic fluid neutrophils are of fetal origin (115), though the subject remains controversial (112). Recently, immunophenotyping of cells isolated from chorioamnionitis-exposed amniotic fluid demonstrated an increased frequency of monocytes/macrophages, B cells, NK cells, and T cells in addition to confirming infiltration of neutrophils (64, 116), suggesting a multifaceted immune infiltrate. The associated inflammatory milieu negatively impacts surfactant composition and function (117), and alters response to therapeutic surfactant in patients with RDS (118). These multifaceted changes occur during the canalicular and saccular stages of lung development (~16–36 wks), and it is tempting to hypothesize that injury at this time may impact alveolarization later in development, as supported by animal data (see below).

## Fetal Systemic Immune Consequences of Chorioamnionitis

In addition to the organ specific manifestations unique to the fetal lung, it is imperative to consider the systemic changes that can occur in response to chorioamnionitis. Of particular importance is the fetal immune system, as the fetal immune response can have a significant impact upon development and contribute to organ dysfunction. In fact, the most well-recognized effect of chorioamnionitis exposure on the neonatal immune system is the elevation of pro-inflammatory cytokines in fetal circulation. IL-6, generally considered the primary signal of fetal immune system activation, is frequently elevated in cord blood (119–121). This connection is notable enough that IL-6 was initially used to define an entity termed fetal inflammatory response syndrome (FIRS), the fetal corollary to adult systemic inflammatory response syndrome (SIRS). Other inflammatory mediators that are frequently elevated include TNFα, IL-1, and IL-8 (122–124). Importantly, while these mediators are significantly increased in infants exposed to severe chorioamnionitis, they are less elevated in mild chorioamnionitis (109, 125). This difference is likely in part due to the classification system of chorioamnionitis, as the histopathologic hallmark associated with FIRS is funisitis (121).

Beyond soluble mediators and histopathologic findings, transcriptional analyses have revealed several chorioamnionitis-induced alterations of the fetal immune system. Whole blood transcriptional analyses revealed ~500 differentially expressed genes in chorioamnionitis compared to non-exposed neonates (126). Although the cellular source of differentially expressed genes was not determined, some of the top altered pathways pointed to activation of innate immune pathways in exposed neonates. In infants with chorioamnionitis, there was an increased proportion of total circulating monocytes (127), as well as neutrophils as far out as 6 days after birth (128). The increased levels of neutrophils could be due to the elevated plasma G-CSF in infants with FIRS (129). Cord blood neutrophils and monocytes in the context of clinical chorioamnionitis were found to have increased expression of TLR4 and TREM-1 (130). It was found that fetal bone marrow monocytes are distinct from adults (131). Besides numerous transcriptional differences observed, fetal monocytes were found to possess enhanced STAT phosphorylation in response in to IFNγ, IL-4, and IL-6 stimulation even at lower concentrations compared to adult monocytes (131). Although the consequences of these differences were not examined, it could suggest that fetal monocytes are highly sensitive to an inflammatory milieu it may encounter. However, analyses of *in vitro*-stimulated monocytes from chorioamnionitis-exposed neonates suggest blunted responsiveness. Indeed, RNAseq analyses of chorioamnionitis-exposed monocytes that were stimulated *in vitro* with *Staphylococcus epidermidis* uncovered a distinct transcriptional profile of hypo-responsiveness (127). In addition, chorioamnionitis exposure in preterm infants has also been shown to increase monocytic H3K4me3 methylation marks, which are tightly associated with inactive gene promoters (132). Monocytes from chorioamnionitis-exposed term infants with increased H3K4me3 modifications produced less IL-1β, IL-6, and IL-8 when stimulated with LPS (132). These data are also in agreement with animal models of chorioamnionitis, which documented that intra-amniotic LPS induces maturation of fetal monocyte function, but long-term or repeated exposures to either LPS or *U. parvum* appear to drive *ex vivo* hypo-responsiveness (133, 134). Together, these data suggest that chorioamnionitis exposure contributes to monocyte dysfunction, with a dissociated phenotype, e.g., enhanced markers of activation directly *ex vivo*, but paradoxically, low response to further stimulation, which could drive the higher risk of sepsis related complications in these infants (127). Dendritic cells (DCs) are another population present in the fetus that are responsive to inflammatory signals such as TLR ligands (135). To our knowledge, their response to chorioamnionitis exposure has not been carefully analyzed, but it is likely that, as for macrophages, the inflammatory mediators present in the AF and the fetal circulation stimulate a fetal DC response that could potentially influence adaptive immunity. In addition, another cell population that intra-uterine inflammation induces is granulocytic myeloid-derived suppressor cells (GR-MDSC) (136), which may participate in chorioamnionitis-induced dysfunction of innate immune responses.

Analysis of adaptive immune responses in chorioamnionitis-exposed infants has mainly focused on T cells, in particular CD4^+^ T cells. Chorioamnionitis exposure has been reported by several groups to drive the emergence of circulating T-effector memory cells (CD4^+^CD25^lo^CD127^hi^), with a Th1/Th17-like phenotype (126, 137–139), although one study did not find such a difference (66). Chorioamnionitis also changed the metabolic profile of CD4^+^ T cells, altering metabolites that are part of the tryptophan catabolism and glutathione detoxification pathway, which could be linked to the enhanced development of a Th1 response (137). Enhanced CD4 production of IL-6 has also been reported (137, 140). The mechanisms driving the presence of activated T cells in the context of chorioamnionitis remain unclear, though a recent report showed increased number of activated maternal alloantigen-responsive T cells in preterm infants (141). These activated T cell appeared independent of chorioamnionitis, but this study did not distinguish mild vs. severe chorioamnionitis, which may explain the overall lack of association.

FoxP3^+^ regulatory T cells (Treg) are an abundant CD4^+^ T cell subset *in utero* (142–144) and are important to inhibit fetal T cell responses against self- and non-self-antigens, including maternal alloantigens (143). Therefore, they have been one of the most studied T cell populations in neonates. However, no consensus has yet been reached on the effect of chorioamnionitis on the frequency of Tregs, as chorioamnionitis has been associated with either no change (140, 145) or decrease (139) in Treg frequency. These discrepancies could be due to the use of different criteria to define chorioamnionitis in different studies. Additionally, difference in the age of preterm infants and the markers used to identify Tregs in each study have complicated interpretation.

There may be additional qualitative differences in the Treg population as a result of exposure to inflammation. Indeed, chorioamnionitis in preterm infants was associated with increased number of Tregs expressing the Th17 main transcription factor RORγt (146) or capable of producing IL-17A (139). Similarly, RORγt/FOXP3 mRNA ratio is increased in the blood of premature infants exposed to severe chorioamnionitis, but not to mild chorioamnionitis (138). Treg suppressive capacity was also diminished in late preterm infants exposed to severe chorioamnionitis (145). However, whether this diminished overall suppressive function is mechanistically due to the increased proportion of inflammatory Tregs remains to be determined.

## Animal Models of Chorioamnionitis

**Figure 1:**
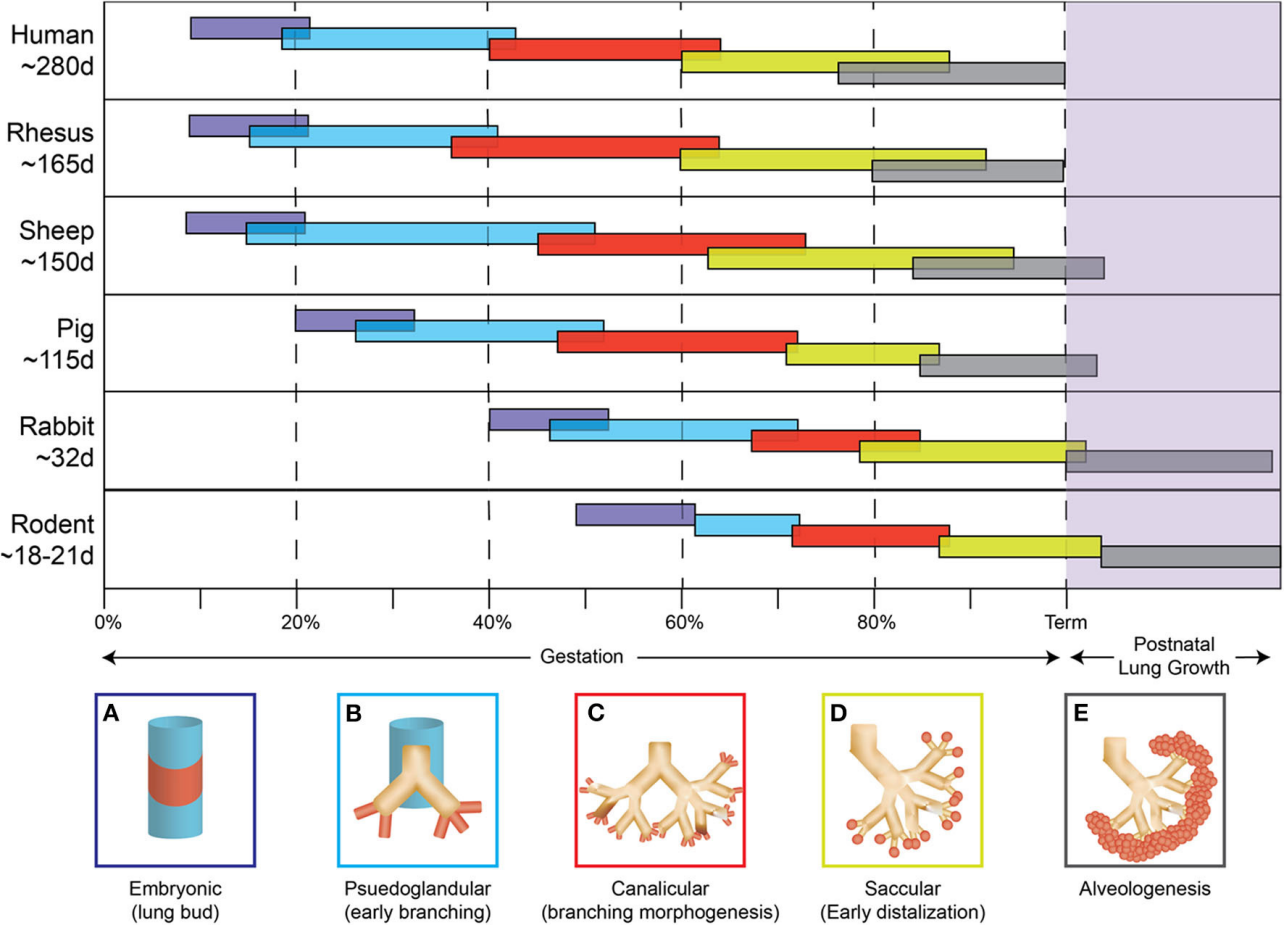
Species comparison of lung development. The five stages of lung development **(A)** embryonic, **(B)** pseudoglandular, **(C)** canalicular, **(D)** saccular, **(E)** alveologenesis during gestation and postnatally are compared between different animal models used to study chorioamnionitis.

**Figure 2:**
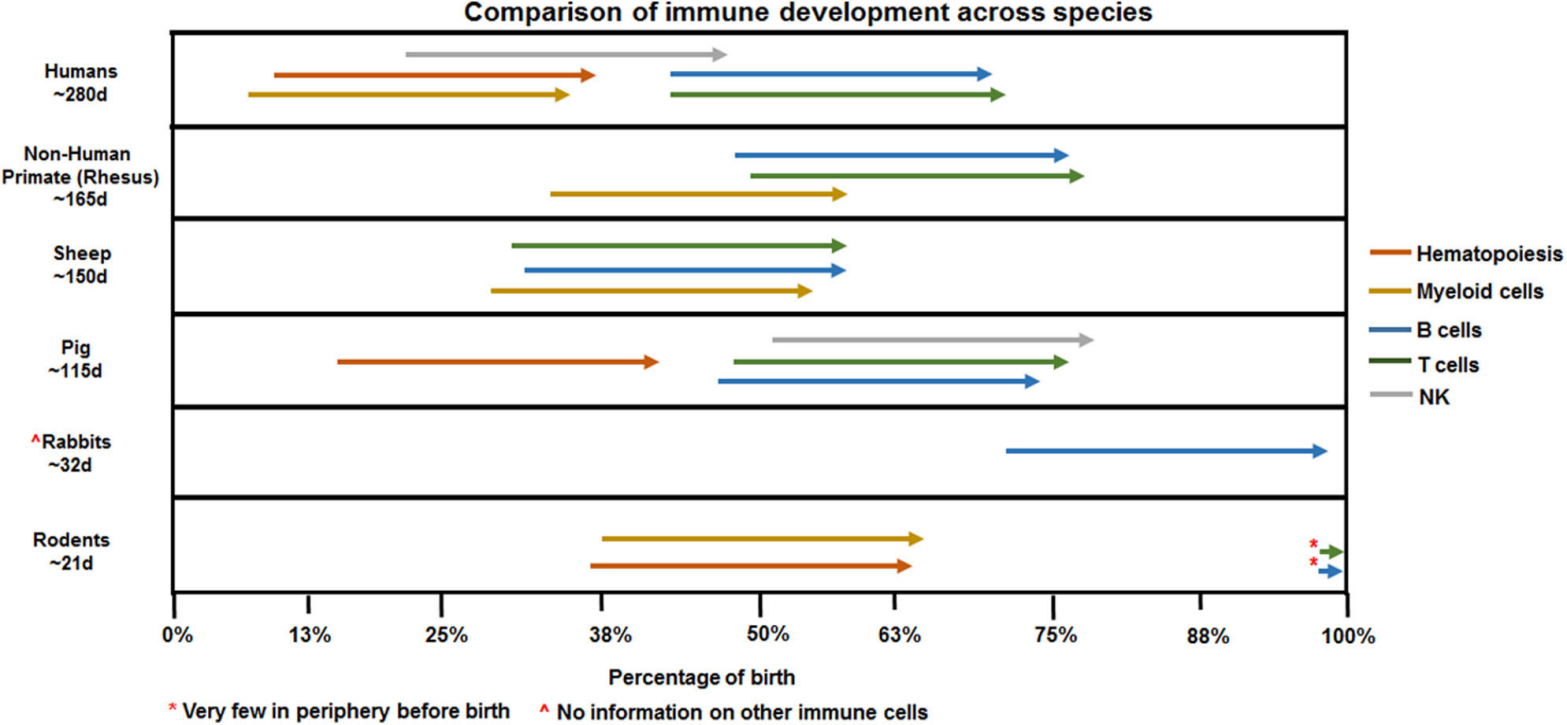
Species comparison of immune ontogeny. Arrows denoting hematopoiesis or macrophages reference presence in AGM/yolk sac (human, rodent, pig) or fetal periphery (non-human primate, sheep). NK, T, and B cell arrows mark appearance in the fetal periphery outside of primary lymphoid organs such as bone marrow (NK, B cells) or thymus (T cells).

**Figure 3:**
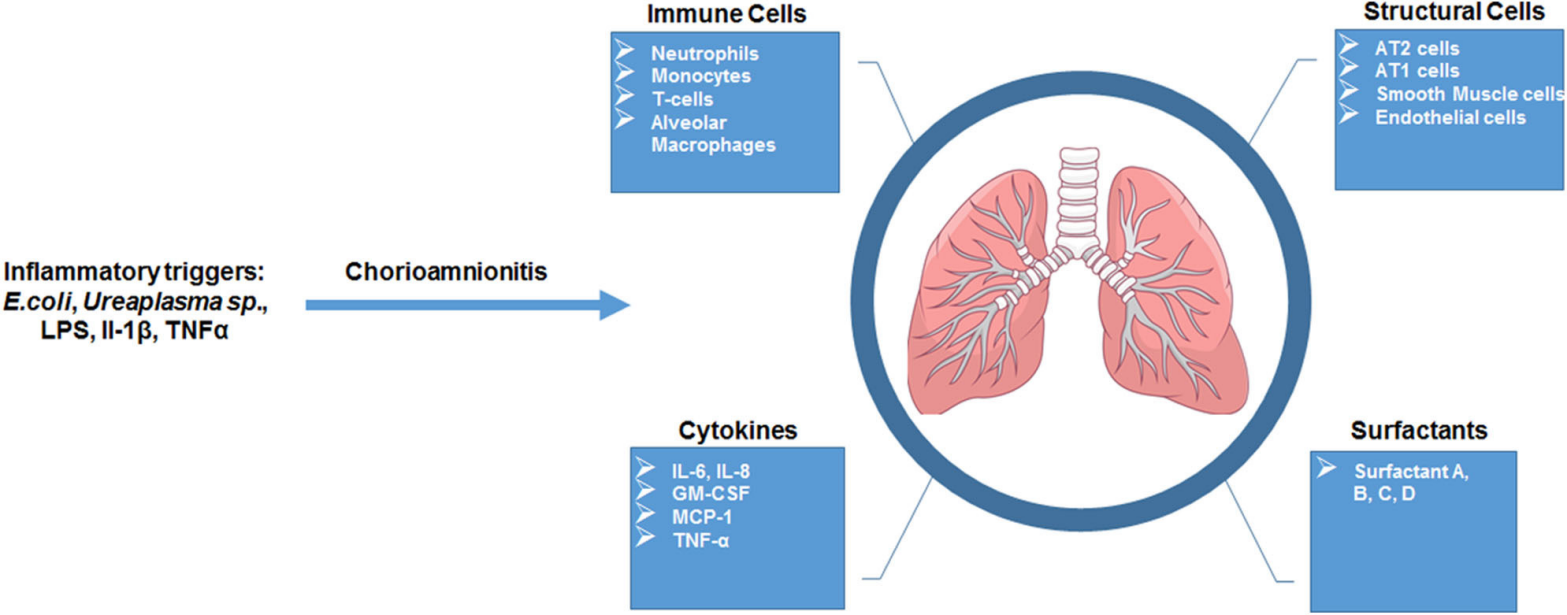
Overview of fetal lung consequences in response to *in utero* inflammatory challenge. Summarized schematic of observations made of the fetal lung response in animal models of chorio.

Studies focused on human neonates have not yet clearly identified actionable mechanisms related to the diagnosis or management of chorioamnionitis. This is due in part to the inherent logistical and ethical limitations of clinical studies as well as notable challenges related to access to fetal organs. In the next section, we will describe the data generated through the currently available animal models of chorioamnionitis (e.g., mouse, rat, rabbit, pig, sheep, and non-human primate) and how they provide a more specific understanding of the pathophysiology of fetal amniotic inflammation (Table 1). Figures 1, 2 summarize our current knowledge on the development of the lung and aspects of the immune system across species, respectively. These studies have shown a wide-ranging effect of chorioamnionitis on a number of organ systems, including the heart, lungs, intestine, brain, eyes, and kidney (119, 121, 148, 150–152). Here, we will focus on what these models have taught us about fetal lung and immune system development, maturation, and activation after fetal inflammation which is summarized in Figure 3.

**Table 1:** Species comparison of animal models of chorioamnionitis to clinical observations in humans.

**Model**	**Human**	**Rhesus macaque**	**Sheep**	**Pig**	**Rabbit**	**Rodent**
Gestational period	280 day	~165 days	~150 days	~115 days	~32 day	~20–22 days
Placenta type	Discord, Hemochorial	Discord, Hemochorial	Cotyledonary, Epitheliochorial	Diffuse, Epitheliochorial	Discord, Hemochorial	Discord, Hemochorial
Agents used to induce chorio	Various microbes and/or mediators (absence of microbes, sterile inflammation) implicated	LPS, IL-1, Ureaplasma, TNFα	LPS, Ureaplasma, TNFα, L-1	*E. coli*, LPS	*E. coli*, LPS, IL-1	*E. coli*, LPS, IL-1
Pros of model		1. Similarity in organ, immmune ontogeny 2. Avalability of reagents	1. Similarity in organ, immune ontogeny	1. Multiple pups per litter	1. Short gestation 2. Multiple pups per litter	1. Short gestation 2. Multiple pups per litter 3. Availability of reagents 4. Different genetic models
Cons of model	Limited access to samples besides cord blood	1. Expensive (housing, maintenance, etc.) 2. Large studies to acquire sample sizes	1. Limited reagents 2. Expensive (housing, maintenance, etc.) 3. Large studies to acquire sample sizes	1. Challenge reaching the pups equally (multiple amniotic sacs) 2. Limited reagents	1. Challenge reaching the pups equally (multiple amniotic sacs) 2. Limited reagent 3. Unknown immunity pre-birth	1. Challenge reaching the pups equally (multiple amniotic sacs) 2. Small size pre-term (technical issues)

*Refs: Furukawa et al. (147); Kallapur et al. (148); Kim et al. (59); Grigsby et al. (149)*.

### Rodents

With their short gestation period (~20 days) and multiple offspring per litter, allowing for large studies, and the possibility of easily introduced genetic modifications, rodent models provide significant benefits to mechanistic studies. However, multiple caveats have limited their usefulness in the study of chorioamnionitis. First, there is a level of uncertainty of whether an inflammatory challenge reaches each pup equally in the setting of large group gestation. Second, the developmental window of the rodent immune system is distinct from humans. Indeed, as shown in Figure 2, hematopoiesis and development of immune cells starts *in utero* at ~5 weeks in humans, and ~day 8 in mice (153, 154). Additionally, organization of secondary lymphoid organs such as the spleen (follicles and T cell zones) occurs *in utero* in humans while it only occurs late in gestation and post-birth in rodents (155–158). Thus, immunological studies in preterm rodents likely will not accurately reflect preterm human neonates.

The timeline of lung development is more similar between rodents and humans, but differences do exist. The majority of lung development occurs *in utero* for both species with the canalicular-saccular phase in mice (~17days—birth) reflecting changes occurring during the 15–38 weeks of gestation in humans (159, 160). However, alveolarization begins during late gestation in humans (36 weeks) and only postnatally in the mouse (post-natal day 4) (4, 161). In addition, studies of fetal lung inflammation are hindered by the small size of rodent pups, which can make extensive lung processing/manipulation technically challenging. Despite these differences, the rodent model has provided important clues on how inflammation impacts fetal and neonatal lung development.

IA injections of LPS and IL-1β in mice (162, 163) and rat (164) as well as endocervical injection of *E. coli* (165) have been used to induce chorioamnionitis in mice. Several characteristic features of human chorioamnionitis are recapitulated in these models, including neutrophilic infiltration in the placental membranes (163–165) and elevated IL-1β and TNFα in the amniotic fluid (162). There were also alterations to the fetal lung including neutrophil invasion, increased cytokine expression, decreased alveolarization (in those sacrificed post birth) with increased number of alveolar type II cells, the main source of surfactant proteins required for alveolar function (162, 165–168). These structural changes mirror those seen in the retrospective human studies reviewed above.

Given the role of IL-1β identified in early studies, novel mouse models have been generated to better understand this relationship mechanistically. Over-expression of human IL-1β in fetal lung epithelium (169) was sufficient to increase neutrophil and macrophage infiltration to the lung and led to thickening of the saccular septa and airway wall, decreased elastin deposition, poor vascularization of developing alveoli, and hyperplasia of bronchial smooth muscle cells and goblet cells (170). These animals also showed decreased septation, resulting in fewer alveoli by post-natal day 7 (170), consistent with data from LPS and *E. coli* treatment models.

The effect of double hits, namely chorioamnionitis and hyperoxia, has also been explored in rodents. Interestingly, moderate hyperoxia improved lung function in rats exposed to intra-amniotic LPS, whereas severe hyperoxia further reduced it, highlighting the concept that the type of post-natal care, notably of post-natal ventilation, will also influence the overall outcome (163).

### Rabbit

Similar to rodents, rabbits constitute a useful model for several reasons including size, multiple kits per litter, and short gestation (~31 days). The 21–29 day age range of rabbits used in preterm studies (171–174) spans the canalicular and saccular phases of lung development, corresponding to ~15–38 weeks in humans (175). Theses similarities in prenatal lung development make the rabbit an attractive model.

Little is known about the status of the rabbit's fetal immune system besides work detailing rabbit B cell biology (176–179). The peripheral blood of post-natal day 1 kits contained lymphocytes, neutrophils, monocytes, eosinophils, and basophils (180). In addition, lymphocytes from the peripheral blood, spleen, and mesenteric lymph node from these kits proliferated in response to ConA stimulation and produced antibodies following immunization (181), suggesting that the fetal immune system of rabbits is more developed at birth than that of rodents.

Intrauterine *E. coli* in rabbits induced neutrophil infiltration and even necrosis in the placenta (171, 174, 182, 183) along with elevated amniotic fluid cytokines (184). Surprisingly, little cell infiltration into the lung was present at 16–30 h post intrauterine *E. coli* (182); another report showed a transient increase of polymorphonuclear neutrophils into the bronchoalveolar lavage fluid (BALF) of infected kits 0–5 days after exposure and confirmed limited cellular infiltration in lung tissue (174). Together, both studies suggest that there is an early, but mild, immune response in the fetal lung, which is less intense than human and mouse data would have predicted. Despite this limited immune response, *E. coli* treatment did lead to altered lung development similar to other models, predominantly compromised alveolarization with decreased secondary septa (173, 174). IA LPS or IL-1α led to neutrophil infiltration in amniotic and bronchoalveolar fluid, increased surfactant expression and enhanced lung compliance (170, 172), suggesting that inflammatory exposure leads to lung maturation in this model despite the mild immune response.

### Pig

The pig is a larger animal, with a longer gestation period (~115 days) and multiple piglets per gestation. Importantly, it is one animal model where the development of the immune system occurs before birth. As early as 30–60 days gestation, innate and adaptive immune cells have been found in umbilical cord and lymphoid organs (185–190). Additionally, the preterm piglets at ~97–106 gestational days (191, 192) closely reflects the saccular phase of humans (~24–38 weeks) (160, 193). However, despite these strengths, very few studies of chorioamnionitis have been conducted in this model.

The IA administration of *E. coli* (194, 195) or LPS (185, 191, 192) leads to leukocyte infiltration and increased pro-inflammatory cytokine expression in the placenta, the amniotic fluid, and the fetal circulation. Following 3 days of LPS exposure, increased CXCL8 and IL-1 expression as well as MPO^+^ cells were found in the fetal lung (191), suggesting longer term exposure to fetal inflammation may mirror the more acute events seen in other, smaller models.

### Sheep

A strong homology in lung architecture, protein structure, growth factors, and immunity between sheep and humans makes the sheep a very relevant model to study lung development and function (196–199). Ovine lung structures are quite comparable to their human counterparts, particularly for epithelial cell distribution, mast cells, and smooth muscle populations (200–202). The sheep lung also contains phagocytic cells that are capable of responding to pathogens. Sheep tracheal explants have shown mucus coverage, mucociliary clearance, and cell structure that are all similar to humans (203–205).

In sheep, IA LPS or IL-1β lead to lung inflammation characterized by cellular infiltration and maturation, and increased cytokine (GM-CSF, IL-6) expression. Similar to rodent models, inflammation improved lung maturation, but reduced alveoli number (134, 206–208). In contrast to the fetal mouse lung that contains more mature monocytes (209), the fetal sheep lung contains very low numbers of alveolar macrophages in absence of inflammation (210). However, akin to human fetal lung, monocyte/macrophages are recruited to the lung after IA LPS (211). In addition, IA LPS triggers GM-CSF expression in the fetal lung, which induces PU.1, a transcription factor responsible for monocyte to macrophage maturation (211). Mechanistically, the robust responses induced by LPS are partially mediated by IL-1β, as co-administration of IL-1RA diminished LPS-induced neutrophil and monocyte infiltration into the BALF, IL-6 expression, and SP-C expression in the lung parenchyma (212).

In contrast to LPS, exposure to live *Ureaplasma* elicits only a mild response in the fetal lung (213). *Ureaplasma* causes infiltration of neutrophils into the lung, but limited monocyte recruitment, and no change in expression of inflammatory cytokines or surfactant proteins (213). Chronic *Ureaplasma* exposure (≥45 days) leads to a more robust cell infiltration into the BALF, with increased lung expression of IL-1β and IL-8 and lung maturation, but does not affect lung alveoli and vascular development (214–217).

### Non-human Primates (NHP)

NHP have several key characteristics that make them a model of choice to study chorioamnionitis. The singleton long gestation along with hemochorial placentation and the endocrine events surrounding parturition in rhesus are similar to human pregnancy (147, 149). Importantly, the cervical and vaginal microflora of the female rhesus are remarkably similar to human [see (218) and references therein]. The anatomic similarity of the rhesus monkey chest wall to the human one generates a functional residual capacity which comprises a similar percentage of total lung capacity (219, 220) comparable to humans. Due to the similarities in the lung function and immunity (221, 222), rhesus macaques have also been used as preclinical models of house dust mite-induced atopic childhood asthma (223–225). Furthermore, analysis of the airway transcriptome in this model demonstrated a large transcriptomic overlap between macaques and humans (226).

IA IL-1β exposure during rhesus gestation cause a robust, neutrophil-dominated cellular infiltration in the lung associated with increased cytokine expression and elevated lung maturation markers like surfactant A, B, C and D, similar to the sheep model (227). There was also a modest increase in the plasma level of glucocorticoids that are known to induce fetal lung maturation (227, 228). IA injection of *Ureaplasma parvum* and *Mycoplasma* lead to colonization of the fetal lung and BALF (229, 230). Initial studies reported acute inflammation following *Ureaplasma* challenge in rhesus lungs (230). However, similar to what has been described in sheep, subsequent studies suggest that IA *U. parvum* causes only a very mild lung phenotype (229). The reasons for these divergent outcomes in primate models remain unknown, as the same *U. parvum* serovar and the same dose was used in both studies. Key differences in study design include different animal colonies, which could have influenced the microbiome of animals prior to injury, as well as the use of catheterized animals only in the first study. Of note, IA *Ureaplasma parvum* followed by post-natal ventilation lead to significant lung inflammation in fetal baboons (231). When observed, inflammation in the rhesus fetal lung was associated with extensive fibrosis, elevated level of α-SMA and TGFβ1, as well as SMAD, IL-1β, and OSM (231).

## Concluding Remarks and Future Perspectives

Histological and clinical chorioamnionitis frequently occur together, though either can be present without the other (68). Despite similar nomenclature, their associated outcomes do not necessarily correlate, and the interchangeable terminology leads to confusion, likely contributing to the mild effect size noted in studies linking chorioamnionitis with respiratory comorbidities. Activation of the fetal inflammatory response in histological chorioamnionitis can potentially influence the development and maturation of the fetal immune system. This *in utero* exposure may “prime” the developing immune system, even in the absence of infection. Such a “priming” results in a more activated and mature immunophenotype, potentially increasing the susceptibility of infants to later childhood diseases, altering their response to vaccination, or contributing to the development of immunopathological disorders. Indeed, funisitis, and activation of fetal inflammatory response were associated with > 2-fold increased risk of developing BPD. It may, therefore, be more instructive to separate clinical chorioamnionitis from histological funisitis. These entities have distinct clinical outcomes and likely activate different physiological pathways (109, 125). We argue that decoupling the “mild” chorioamnionitis from the “severe” chorioamnionitis, and consider separating the analysis of cases including funisitis, may accelerate our understanding of how the fetal inflammatory response directs the maturation of the fetal/neonatal immune response. The relative contribution of chorioamnionitis to RDS and BPD, independent of risk presented by premature birth, needs to be quantified, as the incidence of chorioamnionitis exposure increases with increasingly premature infants (56, 232).

Second, as mentioned earlier, the role of the microbiome remains controversial. Nevertheless, it is possible that the resident microbiome in mothers affects the fetal immune response and its consequences. As the microbiome is quite variable among humans, future studies will need to address whether this variability also modulates the contribution of inflammatory *in utero* exposures on lifelong lung development.

Third, whether unique anatomical position, which brings the fetal lung in direct contact with the inflammatory mediators in the amniotic fluid, results in organ-specific responses is unclear. Whether lung specific alterations in immune cells (135) reflect the systemic changes or are due to local alterations in cytokine milieu needs to be investigated. Furthermore, propagation of the inflammation from the lung to other organs has been suggested in animal models. Indeed, an elegant study where LPS was administered IA, restricted to the lung (tracheal infusion), gut (stomach infusion), or skin (snout occlusion) demonstrated that resultant gut inflammation was induced by either direct contact of the gut or the lung surface (233), evidenced by mild injury in epithelial cell integrity, impaired epithelial differentiation, and loss of ZO-1 along with mild cellular infiltration. This is just an example of a larger question when it comes to FIRS and multi-organ involvement (119, 151); are there potential interactions between organ systems, and under what conditions? These questions therefore represent a priority, and they need to be addressed in relevant animal models.

Finally, the exact mechanism(s) which drive these adverse respiratory outcomes are not yet known, but recent evidence has implied epigenetic alterations in the setting of tobacco exposure, famine, and infections (234–241). Such epigenetic alterations, possibly due to inflammatory milieu or direct toxic effects in the lung, are prime candidates to explain durable, lifelong, and often subtle alterations in disease susceptibility. Despite this provocative connection, direct evidence supporting this hypothesis remains limited. Another potential mechanism is the fact that Th2 immunity appears critical for lung homeostasis in early post-natal period. IL-33 production gradually increases (starting from embryonic day 19 in mice) as a result of mechanical stresses induced by breathing, resulting in mechanical tension on alveolar type-2 cells (242, 243). IL-33 promoted Th2 immunity by directing the recruitment of ILC2 as well as OX40L expression on DCs (243). Since inflammatory cytokines, for example IL-1 and IL-6, limit Th2 responses (244, 245) it is conceivable that the chorioamnionitis-associated pro-inflammatory phenotype may contribute to altered lung development by interrupting the normal lung-shaping Th2-responses, although this possibility remains to be formally tested. Finally, emerging evidence from murine developmental biology literature implies multiple critical waves of lung development, and inflammation at critical times likely impacts these developing lung structural cells alone. All of these possibilities need to be directly evaluated in future studies to precisely target future therapies.

In conclusion, the continued use of animal models is needed to advance our understanding of the various fetal complications due to chorioamnionitis exposure. Depending on the scientific questions asked and context specificities, different animal models will be more or less useful, and future studies need to start integrating the findings. For example, leveraging the similarities between humans and NHP in regard to the close intersection of the lung and the immune system, in combination with the ability to make genetic modifications in rodents, will provide a framework to better understand the impact of severe chorioamnionitis on the developing fetal lung. Then, major findings from animal models will have to be investigated in human neonates. These studies will require extensive longitudinal studies, in which the severity of chorioamnionitis exposure and its intersection with prematurity are well-documented. Clinical pulmonary outcomes need to be carefully monitored in these infants, through repeated questionnaires, high-end imaging and functional assessments. As lung development continues well into the second decade of life, such studies would necessarily require extensive long term follow up to fully characterize the influence of inflammatory *in utero* exposures on lifelong lung development. Only such integrated studies, spanning from animal models to the clinic, can bring enough understanding on how the fetal inflammatory response affects newborn lung maturation, to design new therapeutic strategies aimed at limiting the risk of progressing respiratory diseases in chorioamnionitis-exposed infants.

## Author Contributions

Drafting of manuscript and figures by CJ, SM, AW, CC, and WZ. All authors contributed to the critical review and editing of final manuscript.

## Conflict of Interest

The authors declare that the research was conducted in the absence of any commercial or financial relationships that could be construed as a potential conflict of interest.

## References

[1] Nicholas JK Megha A Ryan MB Zulfiqar AB Jonathan B Austin C Global, regional, and national disability-adjusted life-years (DALYs) for 315 diseases and injuries and healthy life expectancy (HALE), 1990–2015: a systematic analysis for the Global Burden of Disease Study 2015. Lancet. (2016) 388:1603–58. 10.1016/S0140-6736(16)31460-X 27733283 PMC5388857

[2] Wang H Naghavi M Allen C Barber RM Bhutta ZA Carter A. Global, regional, and national life expectancy, all-cause mortality, and cause-specific mortality for 249 causes of death, 1980–2015: a systematic analysis for the Global Burden of Disease Study 2015. Lancet. (2016) 388:1459–544. 10.1016/S0140-6736(16)31012-1 27733281 PMC5388903

[3] Agustí A Hogg JC Update on the Pathogenesis of chronic obstructive pulmonary disease. N Engl J Med. (2019) 381:1248–56. 10.1056/NEJMra1900475 31553836

[4] Nikolic MZ Sun D Rawlins EL. Human lung development: recent progress and new challenges. Development. (2018) 145:dev163485. 10.1242/dev.163485 30111617 PMC6124546

[5] Li J Wang Z Chu Q Jiang K Li J Tang N. The strength of mechanical forces determines the differentiation of alveolar epithelial cells. Dev Cell. (2018) 44:297–312.e5. 10.1016/j.devcel.2018.01.008 29408236

[6] Desai TJ Cardoso WV Growth factors in lung development and disease: friends or foe? Respir Res. (2002) 3:2 10.1186/rr169 11806837 PMC64813

[7] Alapati D Zacharias WJ Hartman HA Rossidis AC Stratigis JD Ahn NJ. *In utero* gene editing for monogenic lung disease. Sci Transl Med. (2019) 11:eaav8375. 10.1126/scitranslmed.aav8375 30996081 PMC6822403

[8] Fletcher C Peto R. The natural history of chronic airflow obstruction. Br Med J. (1977) 1:1645–8. 10.1136/bmj.1.6077.1645 871704 PMC1607732

[9] Barker DJP Osmond C. Infant mortality, childhood nutrition, and ischaemic heart disease in england and wales. Lancet. (1986) 327:1077–81. 10.1016/S0140-6736(86)91340-1 2871345

[10] Barker DJP Osmond C Winter PD Margetts B Simmonds SJ. Weight in infancy and death from ischaemic heart disease. Lancet. (1989) 334:577–80. 10.1016/S0140-6736(89)90710-1 2570282

[11] Hales CN Barker DJ Clark PM Cox LJ Fall C Osmond C. Fetal and infant growth and impaired glucose tolerance at age 64. BMJ. (1991) 303:1019–22. 10.1136/bmj.303.6809.1019 1954451 PMC1671766

[12] Forsdahl A. Living conditions in childhood and subsequent development of risk factors for arteriosclerotic heart disease. The cardiovascular survey in Finnmark 1974-75. J Epidemiol Community Health. (1978) 32:34–7. 10.1136/jech.32.1.34 262586 PMC1087307

[13] Calkins K Devaskar SU. Fetal origins of adult disease. Curr Probl Pediatr Adolesc Health Care. (2011) 41:158–76. 10.1016/j.cppeds.2011.01.001 21684471 PMC4608552

[14] Wadhwa PD Buss C Entringer S Swanson JM. Developmental origins of health and disease: brief history of the approach and current focus on epigenetic mechanisms. Semin Reprod Med. (2009) 27:358–68. 10.1055/s-0029-1237424 19711246 PMC2862635

[15] O'Donnell KJ Meaney MJ. Fetal origins of mental health: the developmental origins of health and disease hypothesis. Am J Psychiatry. (2017) 174:319–28. 10.1176/appi.ajp.2016.16020138 27838934

[16] Harding R Maritz G. Maternal and fetal origins of lung disease in adulthood. Semin Fetal Neonatal Med. (2012) 17:67–72. 10.1016/j.siny.2012.01.005 22277111

[17] Mann SL Wadsworth ME Colley JR. Accumulation of factors influencing respiratory illness in members of a national birth cohort and their offspring. J Epidemiol Community Health. (1992) 46:286–92. 10.1136/jech.46.3.286 1645088 PMC1059569

[18] Stern DA Morgan WJ Wright AL Guerra S Martinez FD. Poor airway function in early infancy and lung function by age 22 years: a non-selective longitudinal cohort study. Lancet Lond Engl. (2007) 370:758–64. 10.1016/S0140-6736(07)61379-8 17765525 PMC2831283

[19] Berry CE Billheimer D Jenkins IC Lu ZJ Stern DA Gerald LB. A Distinct low lung function trajectory from childhood to the fourth decade of life. Am J Resp Crit Care. (2016) 194:607–12. 10.1164/rccm.201604-0753OC 27585385 PMC5027213

[20] Barker DJ Osmond C Golding J Kuh D Wadsworth ME. Growth *In utero*, blood pressure in childhood and adult life, and mortality from cardiovascular disease. BMJ. (1989) 298:564–7. 10.1136/bmj.298.6673.564 2495113 PMC1835925

[21] Barker DJ Godfrey KM Fall C Osmond C Winter PD Shaheen SO. Relation of birth weight and childhood respiratory infection to adult lung function and death from chronic obstructive airways disease. BMJ. (1991) 303:671–5. 10.1136/bmj.303.6804.671 1912913 PMC1670943

[22] Harvey LL Susan EW. Effects of untreated maternal phenylketonuria and hyperphenylalaninemia on the fetus. N Engl J Med. (1983) 309:1269–74. 10.1056/NEJM198311243092101 6633585

[23] Keith FW Colleen A. Relation of prenatal phenylalanine exposure to infant and childhood cognitive outcomes: results from the International Maternal PKU Collaborative Study. Pediatrics. (2003) 112(6 Pt 2):1537–43. 14654661

[24] Richard K William H Harvey L Kim M Reuben M Bobbye R. The maternal phenylketonuria international study: 1984-2002. Pediatrics. (2003) 112(6 Pt 2):1523–9. 14654658

[25] Roger RL Harvey LL Maternal phenylketonuria and hyperphenylalaninemia. N Engl J Med. (1980) 303:1202–8. 10.1056/NEJM198011203032104 7421947

[26] Nenad B Francjan JvS Harvey LL Phenylketonuria. Lancet Lond Engl. (2010) 376:1417–27. 10.1016/S0140-6736(10)60961-0 20971365

[27] Shelley C Galya G Sally Z Caroline M Philip JL. Effects of dietary management of phenylketonuria on long-term cognitive outcome. Arch Dis Child. (2007) 92:213–8. 10.1136/adc.2006.104786 17068073 PMC2083434

[28] Griffiths P Paterson L Harvie A. Neuropsychological effects of subsequent exposure to phenylalanine in adolescents and young adults with eariy-treated phenylketonuria. J Intell Disabil Res. (1995) 39:365–72. 10.1111/j.1365-2788.1995.tb00540.x 8555712

[29] Barker DJP Osmond C Kajantie E Eriksson JG. Growth and chronic disease: findings in the Helsinki Birth Cohort. Ann Hum Biol. (2009) 36:445–58. 10.1080/03014460902980295 19562567

[30] Roseboom TJ van der Meulen JH Osmond C Barker DJ Ravelli AC Schroeder-Tanka JM. Coronary heart disease after prenatal exposure to the Dutch famine, 1944-45. Heart. (2000) 84:595–8. 10.1136/heart.84.6.595 11083734 PMC1729504

[31] Roseboom TJ van der Meulen JHP Ravelli ACJ Osmond C Barker DJP Bleker OP. Effects of prenatal exposure to the Dutch famine on adult disease in later life: an overview. Mol Cell Endocrinol. (2001) 185:93–8. 10.1016/S0303-7207(01)00721-3 11738798

[32] Neelsen S Stratmann T. Effects of prenatal and early life malnutrition: evidence from the Greek famine. J Health Econ. (2011) 30:479–88. 10.1016/j.jhealeco.2011.03.001 21546107

[33] Yu C Wang J Li Y Han X Hu H Wang F. Exposure to the Chinese famine in early life and hypertension prevalence risk in adults. J Hypertens. (2017) 35:63–8. 10.1097/HJH.0000000000001122 27607452

[34] He P Liu L Salas JMI Guo C Cheng Y Chen G. Prenatal malnutrition and adult cognitive impairment: a natural experiment from the 1959–1961 Chinese famine. Br J Nutr. (2018) 120:198–203. 10.1017/S0007114518000958 29720288

[35] de Rooij SR Wouters H Yonker JE Painter RC Roseboom TJ. Prenatal undernutrition and cognitive function in late adulthood. Proc Natl Acad Sci USA. (2010) 107:16881–6. 10.1073/pnas.1009459107 20837515 PMC2947913

[36] Susser M Stein Z. Timing in prenatal nutrition: a reprise of the dutch famine study. Nutr Rev. (1994) 52:84–94. 10.1111/j.1753-4887.1994.tb01395.x 8015751

[37] de Marco R Accordini S Cerveri I Corsico A Sunyer J Neukirch F. An international survey of chronic obstructive pulmonary disease in young adults according to GOLD stages. Thorax. (2004) 59:120–5. 10.1136/thorax.2003.011163 14760151 PMC1746950

[38] Lange P Celli B Agustí A Boje Jensen G Divo M Faner R. Lung-Function trajectories leading to chronic obstructive pulmonary disease. N Engl J Med. (2015) 373:111–22. 10.1056/NEJMoa1411532 26154786

[39] Sears MR Greene JM Willan AR Wiecek EM Taylor DR Flannery EM. A Longitudinal, population-based, cohort study of childhood asthma followed to adulthood. N Engl J Med. (2003) 349:1414–22. 10.1056/NEJMoa022363 14534334

[40] Owens L Laing IA Zhang G Turner S Le Souëf PN. Airway function in infancy is linked to airflow measurements and respiratory symptoms from childhood into adulthood. Pediatr Pulmonol. (2018) 53:1082–8. 10.1002/ppul.24062 29806178

[41] Balmes JR. Household air pollution from domestic combustion of solid fuels and health. J Allergy Clin Immunol. (2019) 143:1979–87. 10.1016/j.jaci.2019.04.016 31176380

[42] Martinez FD. Early-Life origins of chronic obstructive pulmonary disease. N Engl J Med. (2016) 375:871–8. 10.1056/NEJMra1603287 27579637

[43] Frank DG Kiros B Rob M Gauderman WJ Hita V Edward BR. Maternal smoking during pregnancy, environmental tobacco smoke exposure and childhood lung function. Thorax. (2000) 55:271–6. 10.1136/thorax.55.4.271 10722765 PMC1745733

[44] Svanes C Omenaas E Jarvis D Chinn S Gulsvik A Burney P. Parental smoking in childhood and adult obstructive lung disease: results from the European Community Respiratory Health Survey. Thorax. (2004) 59:295–302. 10.1136/thx.2003.009746 15047948 PMC1763798

[45] Schultz ES Hallberg J Andersson N Thacher JD Pershagen G Bellander T. Early life determinants of lung function change from childhood to adolescence. Resp Med. (2018) 139:48–54. 10.1016/j.rmed.2018.04.009 29858001

[46] Urman R McConnell R Islam T Avol EL Lurmann FW Vora H. Associations of children's lung function with ambient air pollution: joint effects of regional and near-roadway pollutants. Thorax. (2014) 69:540–7. 10.1136/thoraxjnl-2012-203159 24253832 PMC4191894

[47] Simpson SJ Logie KM O'Dea CA Banton GL Murray C Wilson AC. Altered lung structure and function in mid-childhood survivors of very preterm birth. Thorax. (2017) 72:702–11. 10.1136/thoraxjnl-2016-208985 28119488

[48] Thunqvist P Gustafsson PM Schultz ES Bellander T Berggren-Broström E Norman M. Lung function at 8 and 16 years after moderate-to-late preterm birth: a prospective cohort study. Pediatrics. (2016) 137:e20152056. 10.1542/peds.2015-2056 27009034

[49] Rona RJ Gulliford MC Chinn S. Effects of prematurity and intrauterine growth on respiratory health and lung function in childhood. Brit Med J. (1993) 306:817–20. 10.1136/bmj.306.6881.817 8490372 PMC1677317

[50] Manuck TA Levy PT Gyamfi-Bannerman C Jobe AH Blaisdell CJ. Prenatal and perinatal determinants of lung health and disease in early life: a national heart, lung, and blood institute workshop report. JAMA Pediatr. (2016) 170:e154577. 10.1001/jamapediatrics.2015.4577 26953657

[51] van Meel ER den Dekker HT Elbert NJ Jansen PW Moll HA Reiss IK. A population-based prospective cohort study examining the influence of early-life respiratory tract infections on school-age lung function and asthma. Thorax. (2018) 73:167–73. 10.1136/thoraxjnl-2017-210149 29101282 PMC6485606

[52] Svanes C Sunyer J Plana E Dharmage S Heinrich J Jarvis D. Early life origins of chronic obstructive pulmonary disease. Thorax. (2010) 65:14–20. 10.1136/thx.2008.112136 19729360

[53] Bui DS Lodge CJ Burgess JA Lowe AJ Perret J Bui MQ. Childhood predictors of lung function trajectories and future COPD risk: a prospective cohort study from the first to the sixth decade of life. Lancet Respir Med. (2018) 6:535–44. 10.1016/S2213-2600(18)30100-0 29628376

[54] Castro-Rodriguez JA Forno E Rodriguez-Martinez CE Celedón JC. Risk and protective factors for childhood asthma: what is the evidence? J Allergy Clin Immunol Pract. (2016) 4:1111–22. 10.1016/j.jaip.2016.05.003 27286779 PMC5107168

[55] Higgins RD Saade G Polin RA Grobman WA Buhimschi IA Watterberg K. Evaluation and management of women and newborns with a maternal diagnosis of chorioamnionitis: summary of a workshop. Obstet Gynecol. (2016) 127:426–36. 10.1097/AOG.0000000000001246 26855098 PMC4764452

[56] Goldenberg RL Hauth JC Andrews WW. Intrauterine infection and preterm delivery. N Engl J Med. (2000) 342:1500–7. 10.1056/NEJM200005183422007 10816189

[57] Goldenberg RL Culhane JF Iams JD Romero R Epidemiology and causes of preterm birth. Lancet. (2008) 371:75–84. 10.1016/S0140-6736(08)60074-4 18177778 PMC7134569

[58] Goldenberg RL Andrews WW Hauth JC. Choriodecidual infection and preterm birth. Nutr Rev. (2002) 60(5 Pt 2):S19–25. 10.1301/00296640260130696 12035853

[59] Kim SY Choi CW Jung E Lee J Lee JA Kim H. Neonatal morbidities associated with histologic chorioamnionitis defined based on the site and extent of inflammation in very low birth weight infants. J Korean Med Sci. (2015) 30:1476–82. 10.3346/jkms.2015.30.10.1476 26425046 PMC4575938

[60] Pacora P Chaiworapongsa T Maymon E Kim YM Gomez R Yoon BH. Funisitis and chorionic vasculitis: the histological counterpart of the fetal inflammatory response syndrome. J Matern Fetal Neonatal Med. (2002) 11:18–25. 10.1080/jmf.11.1.18.25 12380603

[61] Redline RW Faye-Petersen O Heller D Qureshi F Savell V Vogler C. Amniotic infection syndrome: nosology and reproducibility of placental reaction pattern. Pediart Dev Pathol. (2003) 6:435–48. 10.1007/s10024-003-7070-y 14708737

[62] Tita ATN Andrews WW. Diagnosis and management of clinical chorioamnionitis. Clin Perinatol. (2010) 37:339–54. 10.1016/j.clp.2010.02.003 20569811 PMC3008318

[63] Been JV Vanterpool SF de Rooij JDE Rours GIJG Kornelisse RF van Dongen MCJM. A clinical prediction rule for histological chorioamnionitis in preterm newborns. PLoS ONE. (2012) 7:e46217. 10.1371/journal.pone.0046217 23071549 PMC3465298

[64] Gomez-Lopez N Romero R Galaz J Xu Y Panaitescu B Slutsky R Cellular immune responses in amniotic fluid of women with preterm labor and intra-amniotic infection or intra-amniotic inflammation. Am J Reprod Immunol. (2019) 82:e13171 10.1111/aji.13171 31323170 PMC6788958

[65] Luciano AA Yu H Jackson LW Wolfe LA Bernstein HB. Preterm labor and chorioamnionitis are associated with neonatal T cell activation. PLoS ONE. (2011) 6:e16698. 10.1371/journal.pone.0016698 21347427 PMC3035646

[66] Crespo M Martinez DG Cerissi A Rivera-Reyes B Bernstein HB Lederman MM. Neonatal T-cell maturation and homing receptor responses to Toll-like receptor ligands differ from those of adult naive T cells: relationship to prematurity. Pediatr Res. (2012) 71:136–43. 10.1038/pr.2011.26 22258123 PMC3394681

[67] Gibbs RS Duff P. Progress in pathogenesis and management of clinical intraamniotic infection. Am J Obstet Gynecol. (1991) 164(5 Pt 1):1317–26. 10.1016/0002-9378(91)90707-X 2035575

[68] Kim CJ Romero R Chaemsaithong P Chaiyasit N Yoon BH Kim YM. Acute chorioamnionitis and funisitis: definition, pathologic features, and clinical significance. Am J Obstet Gynecol. (2015) 213(4 Suppl):S29–S52. 10.1016/j.ajog.2015.08.040 26428501 PMC4774647

[69] Menon R Taylor RN Fortunato SJ. Chorioamnionitis – A complex pathophysiologic syndrome. Placenta. (2010) 31:113–20. 10.1016/j.placenta.2009.11.012 20031205

[70] Drucilla JR Ann CC Laura ER Andrew BO Theonia KB Lise Carolyn J. Acute histologic chorioamnionitis at term: nearly always noninfectious. PLoS ONE. (2012) 7:e31819. 10.1371/journal.pone.0031819 22412842 PMC3296706

[71] Kyung Joon O Sun Min K Joon-Seok H Eli M Offer E Bogdan P Twenty-four percent of patients with clinical chorioamnionitis in preterm gestations have no evidence of either culture-proven intraamniotic infection or intraamniotic inflammation. Am J Obstet Gynecol. (2017) 216:604.e1–11. 10.1016/j.ajog.2017.02.035 28257964 PMC5769703

[72] Romero R Miranda J Chaemsaithong P Chaiworapongsa T Kusanovic JP Dong Z. Sterile and microbial-associated intra-amniotic inflammation in preterm prelabor rupture of membranes. J Matern Fetal Neona. (2015) 28:1394–409. 10.3109/14767058.2014.958463 25190175 PMC5371030

[73] Romero R Miranda J Chaiworapongsa T Chaemsaithong P Gotsch F Dong Z. Sterile intra-amniotic inflammation in asymptomatic patients with a sonographic short cervix: prevalence and clinical significance. J Matern Fetal Neona. (2015) 28:1343–59. 10.3109/14767058.2014.954243 25123515 PMC4372495

[74] Romero R Miranda J Chaiworapongsa T Korzeniewski SJ Chaemsaithong P Gotsch F. Prevalence and clinical significance of sterile intra-amniotic inflammation in patients with preterm labor and intact membranes. Am J Reprod Immunol. (2014) 72:458–74. 10.1111/aji.12296 25078709 PMC4192099

[75] Combs CA Gravett M Garite TJ Hickok DE Lapidus J Porreco R. Amniotic fluid infection, inflammation, and colonization in preterm labor with intact membranes. Am J Obstet Gynecol. (2014) 210:125.e1–15. 10.1016/j.ajog.2013.11.032 24274987

[76] Prince AL Ma J Kannan PS Alvarez M Gisslen T Harris RA. The placental membrane microbiome is altered among subjects with spontaneous preterm birth with and without chorioamnionitis. Am J Obstet Gynecol. (2016) 214:627.e1–16. 10.1016/j.ajog.2016.01.193 26965447 PMC4909356

[77] DiGiulio DB Romero R Kusanovic JP Gomez R Kim CJ Seok KS. Prevalence and diversity of microbes in the amniotic fluid, the fetal inflammatory response, and pregnancy outcome in women with preterm pre-labor rupture of membranes. Am J Reprod Immunol. (2010) 64:38–57. 10.1111/j.1600-0897.2010.00830.x 20331587 PMC2907911

[78] Yoneda N Yoneda S Niimi H Ueno T Hayashi S Ito M. Polymicrobial amniotic fluid infection with mycoplasma/ureaplasma and other bacteria induces severe intra-amniotic inflammation associated with poor perinatal prognosis in preterm labor. Am J Reprod Immunol. (2016) 75:112–25. 10.1111/aji.12456 26668114

[79] Urushiyama D Suda W Ohnishi E Araki R Kiyoshima C Kurakazu M. Microbiome profile of the amniotic fluid as a predictive biomarker of perinatal outcome. Sci Rep. (2017) 7:12171. 10.1038/s41598-017-11699-8 28939908 PMC5610236

[80] Lauder AP Roche AM Sherrill-Mix S Bailey A Laughlin AL Bittinger K. Comparison of placenta samples with contamination controls does not provide evidence for a distinct placenta microbiota. Microbiome. (2016) 4:29. 10.1186/s40168-016-0172-3 27338728 PMC4917942

[81] Leiby JS McCormick K Sherrill-Mix S Clarke EL Kessler LR Taylor LJ. Lack of detection of a human placenta microbiome in samples from preterm and term deliveries. Microbiome. (2018) 6:196. 10.1186/s40168-018-0575-4 30376898 PMC6208038

[82] Watterberg KL Demers LM Scott SM Murphy S. Chorioamnionitis and early lung inflammation in infants in whom bronchopulmonary dysplasia develops. Pediatrics. (1996) 97:210–5. 8584379

[83] Watterberg KL Demers LM Scott SM Murphy S. Chorioamnionitis and early lung inflammation in infants in whom bronchopulmonary dysplasia develops. Pediatrics. (1996) 97:210–5. 8584379

[84] Plakkal N Soraisham AS Trevenen C Freiheit EA Sauve R. Histological chorioamnionitis and bronchopulmonary dysplasia: a retrospective cohort study. J Perinatol. (2013) 33:441–5. 10.1038/jp.2012.154 23238570

[85] Metcalfe A Lisonkova S Sabr Y Stritzke A Joseph KS. Neonatal respiratory morbidity following exposure to chorioamnionitis. BMC Pediatr. (2017) 17:128. 10.1186/s12887-017-0878-9 28514958 PMC5436447

[86] Prendergast M May C Broughton S Pollina E Milner AD Rafferty GF. Chorioamnionitis, lung function and bronchopulmonary dysplasia in prematurely born infants. Arch Dis Child Fetal Neonatal Ed. (2011) 96:F270–4. 10.1136/adc.2010.189480 21097839

[87] Dessardo NS Mustać E Dessardo S Banac S Peter B Finderle A. Chorioamnionitis and chronic lung disease of prematurity: a path analysis of causality. Amer J Perinatol. (2012) 29:133–40. 10.1055/s-0031-1295654 22147641

[88] Thomas W Speer CP. Chorioamnionitis is essential in the evolution of bronchopulmonary dysplasia – The case in favour. Paediatr Respir Rev. (2014) 15:49–52. 10.1016/j.prrv.2013.09.004 24128984

[89] Lacaze-Masmonteil T. That Chorioamnionitis is a Risk Factor for Bronchopulmonary Dysplasia – The case against. Paediatr Respir Rev. (2014) 15:53–5. 10.1016/j.prrv.2013.09.005 24120077

[90] Sarno L Della Corte L Saccone G Sirico A Raimondi F Zullo F. Histological chorioamnionitis and risk of pulmonary complications in preterm births: a systematic review and Meta-analysis. J Matern Fetal Neonatal Med. (2019) 32:1–10. 10.1080/14767058.2019.1689945 31722581

[91] Jobe AH. Effects of chorioamnionitis on the fetal lung. Clin Perinatol. (2012) 39:441–57. 10.1016/j.clp.2012.06.010 22954262 PMC3437038

[92] Kumar R Yu Y Story RE Pongracic JA Gupta R Pearson C. Prematurity, chorioamnionitis, and the development of recurrent wheezing: a prospective birth cohort study. J Allergy Clin Immunol. (2008) 121:878–84.e6. 10.1016/j.jaci.2008.01.030 18313129 PMC2993062

[93] Sonnenschein-Van Der Voort AMM Arends LR De Jongste JC Annesi-Maesano I Arshad SH Barros H. Preterm birth, infant weight gain, and childhood asthma risk: a meta-analysis of 147,000 European children. J Allergy Clin Immunol. (2014) 133:1317–29. 10.1016/j.jaci.2013.12.1082 24529685 PMC4024198

[94] McDowell KM Jobe AH Fenchel M Hardie WD Gisslen T Young LR. Pulmonary morbidity in infancy after exposure to chorioamnionitis in late preterm infants. Ann Am Thorac Soc. (2016) 13:867–76. 10.1513/AnnalsATS.201507-411OC 27015030 PMC5018922

[95] Charlotte EB Andrew B John RH Sailesh K Lorcan M. Lung consequences in adults born prematurely. Thorax. (2015) 70:574–80. 10.1136/thoraxjnl-2014-206590 25825005

[96] Heli-Kaisa S Marjaana T Marika SL Petteri H Karoliina W Mirjami S. Lung function in very low birth weight adults. Pediatrics. (2015) 136:642–50. 10.1542/peds.2014-2651d 26347433

[97] Maria V Hege HC Emma S Geir EE Ola DR Trond M. Adult respiratory outcomes of extreme preterm birth. a regional cohort study. Ann Am Thorac Soc. (2015) 12:313–22. 10.1513/AnnalsATS.201406-285OC 25616079

[98] Stocks J Hislop A Sonnappa S. Early lung development: lifelong effect on respiratory health and disease. Lancet Respir Med. (2013) 1:728–42. 10.1016/S2213-2600(13)70118-8 24429276

[99] Doyle LW Faber B Callanan C Freezer N Ford GW Davis NM. Bronchopulmonary dysplasia in very low birth weight subjects and lung function in late adolescence. Pediatrics. (2006) 118:108–13. 10.1542/peds.2005-2522 16818555

[100] Gough A Linden M Spence D Patterson CC Halliday HL McGarvey LPA. Impaired lung function and health status in adult survivors of bronchopulmonary dysplasia. Eur Respir J. (2013) 43:808–16. 10.1183/09031936.00039513 23900988

[101] Jessica YI Roberta LK Judy LA Tina VH Paul EM. Understanding the short- and long-term respiratory outcomes of prematurity and bronchopulmonary dysplasia. Am J Resp Crit Care. (2015) 192:134–56. 10.1164/rccm.201412-2142PP 26038806 PMC4532824

[102] Petra UB Jenny H Per T Eva BB Martin A Gunilla A. Lung function development after preterm birth in relation to severity of Bronchopulmonary dysplasia. BMC Pulm Med. (2017) 17:97. 10.1186/s12890-017-0441-3 28666441 PMC5493015

[103] Praprotnik M Gantar IS Lučovnik M Avčin T Krivec U. Respiratory morbidity, lung function and fitness assessment after bronchopulmonary dysplasia. J Perinatol. (2015) 35:1037–42. 10.1038/jp.2015.124 26468933

[104] Schmidt B Cao L Mackensen-Haen S Kendziorra H Klingel K Speer CP. Chorioamnionitis and inflammation of the fetal lung. Am J Obstet Gynecol. (2001) 185:173–7. 10.1067/mob.2001.13321 11483924

[105] Wirbelauer J Schmidt B Klingel K Cao L Lang F Speer CP. Serum and glucocorticoid-inducible kinase in pulmonary tissue of preterm fetuses exposed to chorioamnionitis. Neonatology. (2008) 93:257–62. 10.1159/000111531 18032912

[106] May M Marx A Seidenspinner S Speer CP. Apoptosis and proliferation in lungs of human fetuses exposed to chorioamnionitis. Histopathology. (2004) 45:283–90. 10.1111/j.1365-2559.2004.01936.x 15330807

[107] Yoon BH Romero R Kim CJ Jun JK Gomez R Choi JH. Amniotic fluid interleukin-6: a sensitive test for antenatal diagnosis of acute inflammatory lesions of preterm placenta and prediction of perinatal morbidity. Am J Obstet Gynecol. (1995) 172:960–70. 10.1016/0002-9378(95)90028-4 7892891

[108] Yoon BH Romero R Jun JK Park KH Park JD Ghezzi F. Amniotic fluid cytokines (interleukin-6, tumor necrosis factor-α, interleukin-1β, and interleukin-8) and the risk for the development of bronchopulmonary dysplasia. Am J Obstet Gynecol. (1997) 177:825–30. 10.1016/S0002-9378(97)70276-X 9369827

[109] Revello R Alcaide MJ Dudzik D Abehsera D Bartha JL. Differential amniotic fluid cytokine profile in women with chorioamnionitis with and without funisitis. J Matern Fetal Neonatal Med. (2016) 29:2161–5. 10.3109/14767058.2015.1077512 26372455

[110] Curley AE Sweet DG Thornton CM O'Hara MD Chesshyre E Pizzotti J. Chorioamnionitis and increased neonatal lung lavage fluid matrix metalloproteinase-9 levels: implications for antenatal origins of chronic lung disease. Am J Obstet Gynecol. (2003) 188:871–5. 10.1067/mob.2003.215 12712078

[111] De Dooy J Colpaert C Schuerwegh A Bridts C Van Der Planken M Ieven M. Relationship between Histologic Chorioamnionitis and Early Inflammatory Variables in Blood, Tracheal Aspirates, and Endotracheal Colonization in Preterm Infants. Pediatr Res. (2003) 54:113–9. 10.1203/01.PDR.0000069702.25801.D1 12672904

[112] Gomez-Lopez N Romero R Xu Y Leng Y Garcia-Flores V Miller D Are amniotic fluid neutrophils in women with intraamniotic infection and/or inflammation of fetal or maternal origin? Am J Obstet Gynecol. (2017) 217:693.e1–16. 10.1016/j.ajog.2017.09.013 28964823 PMC5878926

[113] Romero R Ceska M Avila C Mazor M Behnke E Lindley I. Neutrophil attractant/activating peptide-1/interleukin-8 in term and preterm parturition. Am J Obstet Gynecol. (1991) 165(4 Pt 1):813–20. 10.1016/0002-9378(91)90422-N 1951537

[114] Cherouny PH Pankuch GA Romero R Botti JJ Kuhn DC Demers LM Neutrophil attractant/activating peptide-1/interleukin-8: association with histologic chorioamnionitis, preterm delivery, and bioactive amniotic fluid leukoattractants. Am J Obstet Gynecol. (1993) 169:1299–303. 10.1016/0002-9378(93)90297-V 8238198

[115] Sampson JE Theve RP Blatman RN Shipp TD Bianchi DW Ward BE Fetal origin of amniotic fluid polymorphonuclear leukocytes. Am J Obstet Gynecol. (1997) 176:77–81. 10.1016/S0002-9378(97)80015-4 9024093

[116] Gomez-Lopez N Romero R Xu Y Miller D Leng Y Panaitescu B The immunophenotype of amniotic fluid leukocytes in normal and complicated pregnancies. Am J Reprod Immunol. (2018) 79:e12827 10.1111/aji.12827 29500850 PMC5951617

[117] Meyer KC Zimmerman JJ Inflammation and surfactant. Paediatr Respir Rev. (2002) 3:308–14. 10.1016/S1043-6618(02)00212-8 12457601

[118] Been JV Rours IG Kornelisse RF Jonkers F de Krijger RR Zimmermann LJ Chorioamnionitis alters the response to surfactant in preterm infants. J Pediatr. (2010) 156:10–5.e1. 10.1016/j.jpeds.2009.07.044 19833352

[119] Gomez R Romero R Ghezzi F Yoon BH Mazor M Berry SM The fetal inflammatory response syndrome. Am J Obstet Gynecol. (1998) 179:194–202. 10.1016/S0002-9378(98)70272-8 9704787

[120] Naccasha N Hinson R Montag A Ismail M Bentz L Mittendorf R Association between funisitis and elevated interleukin-6 in cord blood. Obstet Gynecol. (2001) 97:220–4. 10.1097/00006250-200102000-00011 11165585

[121] Gotsch F Romero R Kusanovic JP Mazaki-Tovi S Pineles BL Erez O The fetal inflammatory response syndrome. Clin Obstet Gynecol. (2007) 50:652–83. 10.1097/GRF.0b013e31811ebef6 17762416

[122] Kashlan F Smulian J Shen-Schwarz S Anwar M Hiatt M Hegyi T Umbilical vein interleukin 6 and tumor necrosis factor alpha plasma concentrations in the very preterm infant. Pediatr Infect Dis J. (2000) 19:238–43. 10.1097/00006454-200003000-00013 10749467

[123] Dollner H Vatten L Halgunset J Rahimipoor S Austgulen R Histologic chorioamnionitis and umbilical serum levels of pro-inflammatory cytokines and cytokine inhibitors. BJOG. (2002) 109:534–9. 10.1111/j.1471-0528.2002.01028.x 12066943

[124] Mestan K Yu Y Thorsen P Skogstrand K Matoba N Liu X Cord blood biomarkers of the fetal inflammatory response. J Matern Fetal Neona. (2009) 22:379–87. 10.1080/14767050802609759 PMC5001950 19529994

[125] Gisslen T Alvarez M Wells C Soo MT Lambers DS Knox CL Fetal inflammation associated with minimal acute morbidity in moderate/late preterm infants. Arch Dis Child Fetal Neonatal Ed. (2016) 101:F513–9. 10.1136/archdischild-2015-308518 27010018

[126] Weitkamp JH Guthrie SO Wong HR Moldawer LL Baker HV Wynn JL Histological chorioamnionitis shapes the neonatal transcriptomic immune response. Early Hum Dev. (2016) 98:1–6. 10.1016/j.earlhumdev.2016.06.001 27318328 PMC4947555

[127] de Jong E Hancock DG Wells C Richmond P Simmer K Burgner D Exposure to chorioamnionitis alters the monocyte transcriptional response to the neonatal pathogen Staphylococcus epidermidis. Immunol Cell Biol. (2018) 96:792–804. 10.1111/imcb.12037 29533486

[128] Zanardo V Peruzzetto C Trevisanuto D Cavallin F Vedovato S Straface G Relationship between the neonatal white blood cell count and histologic chorioamnionitis in preterm newborns. J Matern Fetal Neonatal Med. (2012) 25:2769–72. 10.3109/14767058.2012.712562 22813065

[129] Chaiworapongsa T Romero R Berry SM Hassan SS Yoon BH Edwin S The role of granulocyte colony-stimulating factor in the neutrophilia observed in the fetal inflammatory response syndrome. J Perinat Med. (2011) 39:653–66. 10.1515/jpm.2011.072 21801092 PMC3382056

[130] Yan H Li H Zhu L Gao J Li P Zhang Z Increased TLR4 and TREM-1 expression on monocytes and neutrophils in preterm birth: further evidence of a proinflammatory state. J Matern Fetal Neonatal Med. (2019) 32:2961–9. 10.1080/14767058.2018.1452903 29534643

[131] Krow-Lucal ER Kim CC Burt TD McCune JM Distinct functional programming of human fetal and adult monocytes. Blood. (2014) 123:1897–904. 10.1182/blood-2013-11-536094 24518760 PMC3962163

[132] Bermick J Gallagher K denDekker A Kunkel S Lukacs N Schaller M. Chorioamnionitis exposure remodels the unique histone modification landscape of neonatal monocytes and alters the expression of immune pathway genes. FEBS J. (2019) 286:82–109. 10.1111/febs.14728 30565411 PMC6326865

[133] Kallapur SG Jobe AH Ball MK Nitsos I Moss TJM Hillman NH Pulmonary and systemic endotoxin tolerance in preterm fetal sheep exposed to chorioamnionitis. J Immunol. (2007) 179:8491–9. 10.4049/jimmunol.179.12.8491 18056396

[134] Kallapur SG Kramer BW Knox CL Berry CA Collins JJ Kemp MW Chronic fetal exposure to Ureaplasma parvum suppresses innate immune responses in sheep. J Immunol. (2011) 187:2688–95. 10.4049/jimmunol.1100779 21784974 PMC3159703

[135] McGovern N Shin A Low G Low D Duan K Yao LJ Human fetal dendritic cells promote prenatal T-cell immune suppression through arginase-2. Nature. (2017) 546:662–6. 10.1038/nature22795 28614294 PMC6588541

[136] Schwarz J Scheckenbach V Kugel H Spring B Pagel J Härtel C Granulocytic myeloid-derived suppressor cells (GR-MDSC) accumulate in cord blood of preterm infants and remain elevated during the neonatal period. Clin Exp Immunol. (2018) 191:328–37. 10.1111/cei.13059 28963753 PMC5801499

[137] Matta P Sherrod SD Marasco CC Moore DJ McLean JA Weitkamp J-H *In utero* exposure to histological chorioamnionitis primes the exometabolomic profiles of preterm CD4(+) T lymphocytes. J Immunol. (2017) 199:3074–85. 10.4049/jimmunol.1601880 28947540 PMC5659751

[138] Jackson CM Wells CB Tabangin ME Meinzen-Derr J Jobe AH Chougnet CA Pro-inflammatory immune responses in leukocytes of premature infants exposed to maternal chorioamnionitis or funisitis. Pediatr Res. (2017) 81:384–90. 10.1038/pr.2016.232 27814345 PMC5309139

[139] Rito DC Viehl LT Buchanan PM Haridas S Koenig JM. Augmented Th17-type immune responses in preterm neonates exposed to histologic chorioamnionitis. Pediatr Res. (2017) 81:639–45. 10.1038/pr.2016.254 27870827 PMC5395318

[140] Misra R Shah S Fowell D Wang H Scheible K Misra S. Preterm cord blood CD4? T cells exhibit increased IL-6 production in chorioamnionitis and decreased CD4? T cells in bronchopulmonary dysplasia. Hum Immunol. (2015) 76:329–38. 10.1016/j.humimm.2015.03.007 25797206 PMC4507273

[141] Frascoli M Coniglio L Witt R Jeanty C Fleck-Derderian S Myers DE. Alloreactive fetal T cells promote uterine contractility in preterm labor via IFN-γ and TNF-α. Sci Transl Med. (2018) 10:eaan2263. 10.1126/scitranslmed.aan2263 29695455 PMC6449052

[142] Cupedo T Nagasawa M Weijer K Blom B Spits H. Development and activation of regulatory T cells in the human fetus. Eur J Immunol. (2005) 35:383–90. 10.1002/eji.200425763 15682453

[143] Mold JE Michaëlsson J Burt TD Muench MO Beckerman KP Busch MP. Maternal alloantigens promote the development of tolerogenic fetal regulatory T cells *in utero*. Science. (2008) 322:1562–5. 10.1126/science.1164511 19056990 PMC2648820

[144] Michaëlsson J Mold JE McCune JM Nixon DF. Regulation of T cell responses in the developing human fetus. J Immunol. (2006) 176:5741–8. 10.4049/jimmunol.176.10.5741 16670279

[145] Rueda CM Wells CB Gisslen T Jobe AH Kallapur SG Chougnet CA. Effect of chorioamnionitis on regulatory T cells in moderate/late preterm neonates. Hum Immunol. (2015) 76:65–73. 10.1016/j.humimm.2014.10.016 25451985 PMC4282957

[146] Singh AM Sherenian MG Kim KY Erickson KA Yang A Mestan K. Fetal cord blood and tissue immune responses to chronic placental inflammation and chorioamnionitis. Allergy Asthma Clin Immunol. (2018) 14:66. 10.1186/s13223-018-0297-y 30473713 PMC6240933

[147] Furukawa S Kuroda Y Sugiyama A. A comparison of the histological structure of the placenta in experimental animals. J Toxicol Pathol. (2014) 27:11–8. 10.1293/tox.2013-0060 24791062 PMC4000068

[148] Kallapur SG Presicce P Rueda CM Jobe AH Chougnet CA. Fetal immune response to chorioamnionitis. Semin Reprod Med. (2014) 32:56–67. 10.1055/s-0033-1361823 24390922 PMC4118297

[149] Grigsby PL. Animal models to study placental development and function throughout normal and dysfunctional human pregnancy. Semin Reprod Med. (2016) 34:11–6. 10.1055/s-0035-1570031 26752715 PMC4799492

[150] Galinsky R Polglase GR Hooper SB Black MJ Moss TJM. The consequences of chorioamnionitis: preterm birth and effects on development. J Pregnancy. (2013) 2013:412831. 10.1155/2013/412831 23533760 PMC3606792

[151] Gantert M Been JV Gavilanes AWD Garnier Y Zimmermann LJI Kramer BW. Chorioamnionitis: a multiorgan disease of the fetus? J Perinatol. (2010) 30:S21–30. 10.1038/jp.2010.96 20877404

[152] Chau V McFadden DE Poskitt KJ Miller SP Chorioamnionitis in the pathogenesis of brain injury in preterm infants. Clin Perinatol. (2014) 41:83–103. 10.1016/j.clp.2013.10.009 24524448

[153] De Kleer I Willems F Lambrecht B Goriely S Ontogeny of myeloid cells. Front Immunol. (2014) 5:423 10.3389/fimmu.2014.00423 25232355 PMC4153297

[154] Holsapple MP West LJ Landreth KS Species comparison of anatomical and functional immune system development. Birth Defects Res B Dev Reprod Toxicol. (2003) 68:321–34. 10.1002/bdrb.10035 14666995

[155] Friedberg SH Weissman IL Lymphoid tissue architecture. II ontogeny of peripheral T and B cells in mice: evidence against peyer's patches as the site of generation of B cells. J Immunol. (1974) 113:1477–92. 4608249

[156] Namikawa R Mizuno T Matsuoka H Fukami H Ueda R Itoh G Ontogenic development of T and B cells and non-lymphoid cells in the white pulp of human spleen. Immunology. (1986) 57:61–9. 3510968 PMC1453873

[157] Steiniger B Ulfig N Riße M Barth PJ Fetal and early post-natal development of the human spleen: from primordial arterial B cell lobules to a non-segmented organ. Histochem Cell Biol. (2007) 128:205–15. 10.1007/s00418-007-0296-4 17624541

[158] Neely HR Flajnik MF Emergence and evolution of secondary lymphoid organs. Annu Rev Cell Dev Biol. (2016) 32:693–711. 10.1146/annurev-cellbio-111315-125306 27362646 PMC5427510

[159] Zoetis T Hurtt ME Species comparison of lung development. Birth Defects Res Dev Reprod Toxicol. (2003) 68:121–4. 10.1002/bdrb.10014 12866703

[160] Lewin G Hurtt ME Pre- and postnatal lung development: an updated species comparison. Birth Defects Res. (2017) 109:1519–39. 10.1002/bdr2.1089 28876535

[161] Schittny JC Development of the lung. Cell Tissue Res. (2017) 367:427–44. 10.1007/s00441-016-2545-0 28144783 PMC5320013

[162] Prince LS Okoh VO Moninger TO Matalon S Lipopolysaccharide increases alveolar type II cell number in fetal mouse lungs through Toll-like receptor 4 and NF-κB. Am J Physiol Lung Cell Mol Physiol. (2004) 287:L999–1006. 10.1152/ajplung.00111.2004 15475494

[163] Bekkering S Limawan Albert P Nguyen Maria U Widiasmoko Lisa K Lu H Pepe S Postnatal inflammation following intrauterine inflammation exacerbates the development of atherosclerosis in ApoE^−/−^ mice. Clin Sci. (2019) 133:1185–96. 10.1042/CS20190141 31088858

[164] Abdulkadir AA Kimimasa T Bell MJ MacPherson TA Keller BB Yanowitz TD Placental inflammation and fetal hemodynamics in a rat model of chorioamnionitis. Pediatr Res. (2010) 68:513–8. 10.1203/PDR.0b013e3181f851ed 20736882

[165] Pan J Zhan C Yuan T Wang W Shen Y Sun Y Effects and molecular mechanisms of intrauterine infection/inflammation on lung development. Respir Res. (2018) 19:93 10.1186/s12931-018-0787-y 29747649 PMC5946538

[166] Prince LS Dieperink HI Okoh VO Fierro-Perez GA Lallone RL Toll-like receptor signaling inhibits structural development of the distal fetal mouse lung. Dev Dyn. (2005) 233:553–61. 10.1002/dvdy.20362 15830384

[167] Nadeau-Vallée M Chin PY Belarbi L Brien MÈ Pundir S Berryer MH Antenatal suppression of IL-1 protects against inflammation-induced fetal injury and improves neonatal and developmental outcomes in mice. J Immunol. (2017) 198:2047–62. 10.4049/jimmunol.1601600 28148737

[168] Hogmalm A Bry M Bry K. Pulmonary IL-1β expression in early life causes permanent changes in lung structure and function in adulthood. Am J Physiol Lung Cell Mol Physiol. (2018) 314:L936–45. 10.1152/ajplung.00256.2017 29446321

[169] Bry K Whitsett JA Lappalainen U. IL-1beta disrupts postnatal lung morphogenesis in the mouse. Am J Respir Cell Mol Biol. (2007) 36:32–42. 10.1165/rcmb.2006-0116OC 16888287 PMC1899307

[170] Bry K Lappalainen U Hallman M. Intraamniotic interleukin-1 accelerates surfactant protein synthesis in fetal rabbits and improves lung stability after premature birth. J Clin Invest. (1997) 99:2992–9. 10.1172/JCI119494 9185523 PMC508151

[171] Dombroski RA Woodard DS Harper MJK Gibbs RS. A rabbit model for bacteria-induced preterm pregnancy loss. Am J Obstet Gynecol. (1990) 163(6 Part 1):1938–43. 10.1016/0002-9378(90)90777-5 2256505

[172] Bry K Lappalainen U. Intra-amniotic endotoxin accelerates lung maturation in fetal rabbits. Acta Paediatrica. (2001) 90:74–80. 10.1111/j.1651-2227.2001.tb00259.x 11227339

[173] Joram N Launay E Roze JC Caillon J Franco-Montoya ML Bourbon J. Betamethasone worsens chorioamnionitis-related lung development impairment in rabbits. Amer J Perinatol. (2011) 28:605–12. 10.1055/s-0031-1276734 21494996

[174] Gras-Le Guen C Denis C Franco-Montoya ML Jarry A Delacourt C Potel G. Antenatal infection in the rabbit impairs post-natal growth and lung alveolarisation. Eur Respir J. (2008) 32:1520–8. 10.1183/09031936.00023708 18684851

[175] Karnak I Müftüoglu S Çakar N Tanyel FC. Organ growth and lung maturation in rabbit fetuses. Res Exp Med. (1999) 198:277–87. 10.1007/s004330050111 10209763

[176] Tunyaplin C Knight KL. Fetal VDJ gene repertoire in rabbit: evidence for preferential rearrangement of VH1. Eur J Immunol. (1995) 25:2583–7. 10.1002/eji.1830250927 7589130

[177] Mage RG Lanning D Knight KL. B cell and antibody repertoire development in rabbits: the requirement of gut-associated lymphoid tissues. Dev Comp Immunol. (2006) 30:137–53. 10.1016/j.dci.2005.06.017 16098588

[178] Weber J Peng H Rader C. From rabbit antibody repertoires to rabbit monoclonal antibodies. Exp Mol Med. (2017) 49:e305. 10.1038/emm.2017.23 28336958 PMC5382564

[179] Hayward AR Simons MA Lawton AR Mage RG Cooper MD. Pre-B and B cells in rabbits. Ontogeny and allelic exclusion of kappa light chain genes. J Exp Med. (1978) 148:1367–77. 10.1084/jem.148.5.1367 102726 PMC2185044

[180] Jeklova E Leva L Knotigova P Faldyna M. Age-related changes in selected haematology parameters in rabbits. Res Vet Sci. (2009) 86:525–8. 10.1016/j.rvsc.2008.10.007 19041105

[181] Jeklova E Leva L Kudlackova H Faldyna M. Functional development of immune response in rabbits. Vet Immunol Immunopathol. (2007) 118:221–8. 10.1016/j.vetimm.2007.05.003 17614140

[182] Davies JK Shikes RH Sze CI Leslie KK McDuffie RS Romero R. Histologic inflammation in the maternal and fetal compartments in a rabbit model of acute intra-amniotic infection. Am J Obstet Gynecol. (2000) 183:1088–93. 10.1067/mob.2000.108888 11084546

[183] McDuffie RS Blanton SJ Shikes RH Gibbs MDRS. A rabbit model for bacterially induced preterm pregnancy loss: intervention studies with ampicillin-sulbactam. Am J Obstet Gynecol. (1991) 165(5 Part 1):1568–74. 10.1016/0002-9378(91)90406-H 1957892

[184] McDuffie R Sherman MP Gibbs RS. Amniotic fluid tumor necrosis factor-α and interleukin-1 in a rabbit model of bacterially induced preterm pregnancy loss. Am J Obstet Gynecol. (1992) 167:1583–8. 10.1016/0002-9378(92)91745-V 1471670

[185] Trebichavský I Tlaskalová H Cukrowska B Šplíchal I Šinkora J Øeháková Z. Early ontogeny of immune cells and their functions in the fetal pig. Vet Immunol Immunopathol. (1996) 54:75–81. 10.1016/S0165-2427(96)05707-8 8988850

[186] Rehakova Z Trebichavsky I Sinkora J Splichal I Sinkora M. Early ontogeny of monocytes and macrophages in the pig. Physiol Res. (1998) 47:357–63. 10052605

[187] Sinkora J Rehakova Z Sinkora M Cukrowska B Tlaskalova-Hogenova H Early development of immune system in pigs. Vet Immunol Immunopathol. (2002) 87:301–6. 10.1016/S0165-2427(02)00056-9 12072250

[188] Sinkora M Sinkora J Reháková Z Splíchal I Yang H Parkhouse RM. Prenatal ontogeny of lymphocyte subpopulations in pigs. Immunology. (1998) 95:595–603. 10.1046/j.1365-2567.1998.00641.x 9893051 PMC1364358

[189] Šinkora M Butler JE. The ontogeny of the porcine immune system. Dev Comp Immunol. (2009) 33:273–83. 10.1016/j.dci.2008.07.011 18762210 PMC7103207

[190] Maddox JF Mackay CR Brandon MR Ontogeny of ovine lymphocytes. II. An immunohistological study on the development of T lymphocytes in the sheep fetal spleen. Immunology. (1987) 62:107–12. 3308689 PMC1453735

[191] Nguyen DN Thymann T Goericke-Pesch SK Ren S Wei W Skovgaard K. Prenatal intra-amniotic endotoxin induces fetal gut and lung immune responses and postnatal systemic inflammation in preterm pigs. Am J Pathol. (2018) 188:2629–43. 10.1016/j.ajpath.2018.07.020 30314768

[192] Ren S Hui Y Goericke-Pesch S Pankratova S Kot W Pan X. Gut and immune effects of bioactive milk factors in preterm pigs exposed to prenatal inflammation. Am J Physiol Gastrointest Liver Physiol. (2019) 317:G67–77. 10.1152/ajpgi.00042.2019 31091150

[193] Caminita F Merwe Mvd Hance B Krishnan R Miller S Buddington K. A preterm pig model of lung immaturity and spontaneous infant respiratory distress syndrome. Am J Physiol Lung Cell Mol Physiol. (2015) 308:L118–29. 10.1152/ajplung.00173.2014 25398985

[194] Šplíchalová A Trebichavský I Muneta Y Mori Y Šplíchal I. Effect of bacterial virulence on IL-18 expression in the amnion infected with *Escherichia coli*. Am J Reprod Immunol. (2005) 53:255–60. 10.1111/j.1600-0897.2005.00273.x 15833104

[195] Šplíchalová A Šplíchal I Trebichavský I Hojná H Expression of inflammatory markers in pig amnion after intraamniotic infection with nonpathogenic or enteropathogenic *Escherichia coli*. Folia Microbiologica. (2004) 49:751–6. 10.1007/BF02931560 15881414

[196] Meeusen EN Snibson KJ Hirst SJ Bischof RJ Sheep as a model species for the study and treatment of human asthma and other respiratory diseases. Drug Discov Today. (2009) 6:101–6. 10.1016/j.ddmod.2009.12.002

[197] Alsalami M Filippich L. Haematology of foetal sheep. Aust Vet J. (1999) 77:588–94. 10.1111/j.1751-0813.1999.tb13197.x 10561794

[198] Press CM Hein WR Landsverk T. Ontogeny of leucocyte populations in the spleen of fetal lambs with emphasis on the early prominence of B cells. Immunology. (1993) 80:598–604. 7508421 PMC1422258

[199] Sow FB Gallup JM Derscheid R Krishnan S Ackermann MR. Ontogeny of the immune response in the ovine lung. Immunol Invest. (2012) 41:304–16. 10.3109/08820139.2011.631657 22122502 PMC3812944

[200] Plopper CG Mariassy AT Wilson DW Alley JL Nishio SJ Nettesheim P. Comparison of nonciliated tracheal epithelial cells in six mammalian species: ultrastructure and population densities. Exp Lung Res. (1983) 5:281–94. 10.3109/01902148309061521 6662075

[201] Miller HR. Mucosal mast cells and the allergic response against nematode parasites. Vet Immunol Immunopathol. (1996) 54:331–6. 10.1016/S0165-2427(96)05696-6 8988878

[202] Collie DD Pyrah I Watt NJ. Distribution and quantitation of lung parenchymal contractile tissue in ovine lentivirus-induced lymphoid interstitial pneumonia. Do tissue forces limit lung distensibility? Lab Invest. (1995) 73:441–7. 7564278

[203] Ferrari S Kitson C Farley R Steel R Marriott C Parkins DA. Mucus altering agents as adjuncts for nonviral gene transfer to airway epithelium. Gene Ther. (2001) 8:1380–6. 10.1038/sj.gt.3301525 11571577

[204] French AT Bethune JA Knight PA McNeilly TN Wattegedera S Rhind S. The expression of intelectin in sheep goblet cells and upregulation by interleukin-4. Vet Immunol Immunopathol. (2007) 120:41–6. 10.1016/j.vetimm.2007.07.014 17727963

[205] Abeynaike L Meeusen EN Bischof RJ. An ovine tracheal explant culture model for allergic airway inflammation. J Inflamm. (2010) 7:46. 10.1186/1476-9255-7-46 20804555 PMC2940870

[206] Willet KE Kramer BW Kallapur SG Ikegami M Newnham JP Moss TJ. Intra-amniotic injection of IL-1 induces inflammation and maturation in fetal sheep lung. Am J Physiol Lung Cell Mol Physiol. (2002) 282:L411–20. 10.1152/ajplung.00097.2001 11839534

[207] Willet KE Jobe AH Ikegami M Newnham J Sly PD. Pulmonary interstitial emphysema 24 hours after antenatal betamethasone treatment in preterm sheep. Am J Respir Crit Care Med. (2000) 162(3 Pt 1):1087–94. 10.1164/ajrccm.162.3.9906103 10988135

[208] Bachurski CJ Ross GF Ikegami M Kramer BW Jobe AH. Intra-amniotic endotoxin increases pulmonary surfactant proteins and induces SP-B processing in fetal sheep. Am J Physiol Lung Cell Mol Physiol. (2001) 280:L279–85. 10.1152/ajplung.2001.280.2.L279 11159007

[209] Blackwell TS Hipps AN Yamamoto Y Han W Barham WJ Ostrowski MC NF-κB signaling in fetal lung macrophages disrupts airway morphogenesis. J Immunol. (2011) 187:2740–7. 10.4049/jimmunol.1101495 21775686 PMC3159744

[210] Kallapur SG Kramer BW Moss TJ Newnham JP Jobe AH Ikegami M. Maternal glucocorticoids increase endotoxin-induced lung inflammation in preterm lambs. Am J Physiol Lung Cell Mol Physiol. (2003) 284:L633–42. 10.1152/ajplung.00344.2002 12471018

[211] Kramer BW Joshi SN Moss TJ Newnham JP Sindelar R Jobe AH. Endotoxin-induced maturation of monocytes in preterm fetal sheep lung. Am J Physiol Lung Cell Mol Physiol. (2007) 293:L345–53. 10.1152/ajplung.00003.2007 17513458

[212] Kallapur SG Moss TJ Auten RL Jr Nitsos I Pillow JJ Kramer BW. IL-8 signaling does not mediate intra-amniotic LPS-induced inflammation and maturation in preterm fetal lamb lung. Am J Physiol Lung Cell Mol Physiol. (2009) 297:L512–9. 10.1152/ajplung.00105.2009 19574422 PMC2739771

[213] Collins JJ Kallapur SG Knox CL Nitsos I Polglase GR Pillow JJ. Inflammation in fetal sheep from intra-amniotic injection of Ureaplasma parvum. Am J Physiol Lung Cell Mol Physiol. (2010) 299:L852–60. 10.1152/ajplung.00183.2010 20935228 PMC3006269

[214] Moss TJ Nitsos I Knox CL Polglase GR Kallapur SG Ikegami M. Ureaplasma colonization of amniotic fluid and efficacy of antenatal corticosteroids for preterm lung maturation in sheep. Am J Obstet Gynecol. (2009) 200:96.e1–6. 10.1016/j.ajog.2008.08.044 19121661 PMC2637947

[215] Moss TJ Knox CL Kallapur SG Nitsos I Theodoropoulos C Newnham JP Experimental amniotic fluid infection in sheep: effects of Ureaplasma parvum serovars 3 and 6 on preterm or term fetal sheep. Am J Obstet Gynecol. (2008) 198:122.e1–8. 10.1016/j.ajog.2007.06.065 18166324 PMC2213425

[216] Polglase GR Dalton RG Nitsos I Knox CL Pillow JJ Jobe AH. Pulmonary vascular and alveolar development in preterm lambs chronically colonized with Ureaplasma parvum. Am J Physiol Lung Cell Mol Physiol. (2010) 299:L232–41. 10.1152/ajplung.00369.2009 20495079 PMC2928606

[217] Moss TJ Nitsos I Ikegami M Jobe AH Newnham JP. Experimental intrauterine Ureaplasma infection in sheep. Am J Obstet Gynecol. (2005) 192:1179–86. 10.1016/j.ajog.2004.11.063 15846199

[218] Gravett MG Witkin SS Haluska GJ Edwards JL Cook MJ Novy MJ. An experimental model for intraamniotic infection and preterm labor in rhesus monkeys. Am J Obstet Gynecol. (1994) 171:1660–7. 10.1016/0002-9378(94)90418-9 7802084

[219] Koike E Kobayashi T Nelson DJ McWilliam AS Holt PG. Effect of ozone exposure on alveolar macrophage-mediated immunosuppressive activity in rats. Toxicol Sci. (1998) 41:217–23. 10.1093/toxsci/41.2.217 9520358

[220] Pare PD Boucher R Michoud MC Hogg JC. Static lung mechanics of intact and excised rhesus monkey lungs and lobes. J Appl Physiol Respir Environ Exerc Physiol. (1978) 44:547–52. 10.1152/jappl.1978.44.4.547 417053

[221] Batchelder CA Duru N Lee CI Baker CAR Swainson L McCune JM. Myeloid-lymphoid ontogeny in the rhesus monkey (Macaca mulatta). Anat Rec. (2014) 297:1392–406. 10.1002/ar.22943 24867874 PMC4120262

[222] Hendrickx AG Makori N Peterson P. The nonhuman primate as a model of developmental immunotoxicity. Hum Exp Toxicol. (2002) 21:537–42. 10.1191/0960327102ht294oa 12458913

[223] Fanucchi MV Schelegle ES Baker GL Evans MJ McDonald RJ Gershwin LJ. Immunostimulatory oligonucleotides attenuate airways remodeling in allergic monkeys. Am J Respir Crit Care Med. (2004) 170:1153–7. 10.1164/rccm.200404-533OC 15306532 PMC3927836

[224] Seshasayee D Lee WP Zhou M Shu J Suto E Zhang J. *In vivo* blockade of OX40 ligand inhibits thymic stromal lymphopoietin driven atopic inflammation. J Clin Invest. (2007) 117:3868–78. 10.1172/JCI33559 18060034 PMC2096422

[225] Plopper CG Joad JP Miller LA Schelegle ES Fanucchi MV Van Winkle LS. Lung effects of inhaled corticosteroids in a rhesus monkey model of childhood asthma. Clin Exp Allergy. (2012) 42:1104–18. 10.1111/j.1365-2222.2012.04005.x 22702509 PMC3913647

[226] Abbas AR Jackman JK Bullens SL Davis SM Choy DF Fedorowicz G. Lung gene expression in a rhesus allergic asthma model correlates with physiologic parameters of disease and exhibits common and distinct pathways with human asthma and a mouse asthma model. Am J Pathol. (2011) 179:1667–80. 10.1016/j.ajpath.2011.06.009 21819959 PMC3181391

[227] Kallapur SG Presicce P Senthamaraikannan P Alvarez M Tarantal AF Miller LM. Intra-amniotic IL-1β induces fetal inflammation in rhesus monkeys and alters the regulatory T cell/IL-17 balance. J Immunol. (2013) 191:1102–9. 10.4049/jimmunol.1300270 23794628 PMC3720768

[228] Jobe AH Kallapur SG Kramer BW Chapter 3 - perinatal events and their influence on lung development and function. In: Bancalari E editor. The Newborn Lung: Neonatology Questions and Controversies, 2nd Edn Philadelphia: W.B. Saunders (2012). p. 57–89. 10.1016/B978-1-4377-2682-4.00003-2

[229] Senthamaraikannan P Presicce P Rueda CM Maneenil G Schmidt AF Miller LA. Intra-amniotic Ureaplasma parvum-induced maternal and fetal inflammation and immune responses in rhesus macaques. J Infect Dis. (2016) 214:1597–604. 10.1093/infdis/jiw408 27601620 PMC6392471

[230] Novy MJ Duffy L Axthelm MK Sadowsky DW Witkin SS Gravett MG. Ureaplasma parvum or Mycoplasma hominis as sole pathogens cause chorioamnionitis, preterm delivery, and fetal pneumonia in rhesus macaques. Reprod Sci. (2009) 16:56–70. 10.1177/1933719108325508 19122105

[231] Viscardi RM Atamas SP Luzina IG Hasday JD He JR Sime PJ. Antenatal Ureaplasma urealyticum respiratory tract infection stimulates proinflammatory, profibrotic responses in the preterm baboon lung. Pediatr Res. (2006) 60:141–6. 10.1203/01.pdr.0000228322.73777.05 16864693

[232] Romero R Espinoza J Gonçalves LF Kusanovic JP Friel L Hassan S. The role of inflammation and infection in preterm birth. Semin Reprod Med. (2007) 25:21–39. 10.1055/s-2006-956773 17205421 PMC8324073

[233] Wolfs TG Kramer BW Thuijls G Kemp MW Saito M Willems MG Chorioamnionitis-induced fetal gut injury is mediated by direct gut exposure of inflammatory mediators or by lung inflammation. Am J Physiol Gastrointest Liver Physiol. (2014) 306:G382–93. 10.1152/ajpgi.00260.2013 24458021 PMC3949018

[234] Winden DR Barton DB Betteridge BC Bodine JS Jones CM Rogers GD. Antenatal exposure of maternal secondhand smoke (SHS) increases fetal lung expression of RAGE and induces RAGE-mediated pulmonary inflammation. Respir Res. (2014) 15:129. 10.1186/s12931-014-0129-7 25338658 PMC4207891

[235] Bauer T Trump S Ishaque N Thürmann L Gu L Bauer M. Environment-induced epigenetic reprogramming in genomic regulatory elements in smoking mothers and their children. Mol Syst Biol. (2016) 12:861. 10.15252/msb.20156520 27013061 PMC4812527

[236] Gonzalez-Nahm S Mendez MA Benjamin-Neelon SE Murphy SK Hogan VK Rowley DL. DNA methylation of imprinted genes at birth is associated with child weight status at birth, 1 year, and 3 years. Clin Epigenetics. (2018) 10:90. 10.1186/s13148-018-0521-0 29988473 PMC6025828

[237] Elmar WT Jelle JG Ramin M Hongcang G Hein P Yanju Z DNA methylation signatures link prenatal famine exposure to growth and metabolism. Nat Commun. (2014) 5:5592 10.1038/ncomms6592 25424739 PMC4246417

[238] Elmar WT Lumey LH Rudolf PT Dennis K Hein P Aryeh DS. DNA methylation differences after exposure to prenatal famine are common and timing- and sex-specific. Hum Mol Genet. (2009) 18:4046–53. 10.1093/hmg/ddp353 19656776 PMC2758137

[239] Heijmans BT Tobi EW Stein AD Putter H Blauw GJ Susser ES. Persistent epigenetic differences associated with prenatal exposure to famine in humans. Proc National Acad Sci USA. (2008) 105:17046–9. 10.1073/pnas.0806560105 18955703 PMC2579375

[240] Luqi S Changwei L Zhenghe W Ruiyuan Z Ye S Toni M. Early-life exposure to severe famine is associated with higher methylation level in the IGF2 gene and higher total cholesterol in late adulthood: the Genomic Research of the Chinese Famine (GRECF) study. Clin Epigenetics. (2019) 11:88. 10.1186/s13148-019-0676-3 31182144 PMC6558811

[241] Lumey LH Mary Beth T Lissette DC Yuyan L Qiao W Ezra S. Adult global DNA methylation in relation to pre-natal nutrition. Int J Epidemiol. (2012) 41:116–23. 10.1093/ije/dyr137 22422450 PMC3304521

[242] Saluzzo S Gorki AD Rana BMJ Martins R Scanlon S Starkl P. First-Breath-induced type 2 pathways shape the lung immune environment. Cell Rep. (2017) 18:1893–905. 10.1016/j.celrep.2017.01.071 28228256 PMC5329122

[243] De Kleer IM Kool M De Bruijn MJW Willart M Van Moorleghem J Schuijs MJ. Perinatal activation of the Interleukin-33 pathway promotes type 2 immunity in the developing lung. Immunity. (2016) 45:1285–98. 10.1016/j.immuni.2016.10.031 27939673

[244] Coomes SM Pelly VS Kannan Y Okoye IS Czieso S Entwistle LJ. IFNγ and IL-12 Restrict Th2 responses during helminth/plasmodium co-infection and promote IFNγ from Th2 cells. PLoS Pathog. (2015) 11:e1004994. 10.1371/journal.ppat.1004994 26147567 PMC4493106

[245] Ahmed N French T Rausch S Kuhl A Hemminger K Dunay IR. Toxoplasma co-infection prevents Th2 differentiation and leads to a helminth-specific Th1 response. Front Cell Infect Microbiol. (2017) 7:341. 10.3389/fcimb.2017.00341 28791259 PMC5524676

